# Multi-scale spiking network model of human cerebral cortex

**DOI:** 10.1093/cercor/bhae409

**Published:** 2024-10-18

**Authors:** Jari Pronold, Alexander van Meegen, Renan O Shimoura, Hannah Vollenbröker, Mario Senden, Claus C Hilgetag, Rembrandt Bakker, Sacha J van Albada

**Affiliations:** Institute for Advanced Simulation (IAS-6), Jülich Research Centre, D-52428 Jülich, Germany; RWTH Aachen University, D-52062 Aachen, Germany; Institute for Advanced Simulation (IAS-6), Jülich Research Centre, D-52428 Jülich, Germany; Institute of Zoology, University of Cologne, D-50674 Cologne, Germany; Institute for Advanced Simulation (IAS-6), Jülich Research Centre, D-52428 Jülich, Germany; Institute for Advanced Simulation (IAS-6), Jülich Research Centre, D-52428 Jülich, Germany; Heinrich Heine University Düsseldorf, D-40225 Düsseldorf, Germany; Faculty of Psychology and Neuroscience, Department of Cognitive Neuroscience, Maastricht University, NL-6229 ER Maastricht, The Netherlands; Faculty of Psychology and Neuroscience, Maastricht Brain Imaging Centre, Maastricht University, NL-6229 ER Maastricht, The Netherlands; Institute of Computational Neuroscience, University Medical Center Eppendorf, Hamburg University, D-20246 Hamburg, Germany; Institute for Advanced Simulation (IAS-6), Jülich Research Centre, D-52428 Jülich, Germany; Donders Institute for Brain, Cognition and Behavior, Radboud University Nijmegen, NL-6525 EN Nijmegen, The Netherlands; Institute for Advanced Simulation (IAS-6), Jülich Research Centre, D-52428 Jülich, Germany; Institute of Zoology, University of Cologne, D-50674 Cologne, Germany

**Keywords:** connectivity, large-scale, neural network, resting-state activity, simulation

## Abstract

Although the structure of cortical networks provides the necessary substrate for their neuronal activity, the structure alone does not suffice to understand the activity. Leveraging the increasing availability of human data, we developed a multi-scale, spiking network model of human cortex to investigate the relationship between structure and dynamics. In this model, each area in one hemisphere of the Desikan–Killiany parcellation is represented by a $1\,\mathrm{mm^{2}}$ column with a layered structure. The model aggregates data across multiple modalities, including electron microscopy, electrophysiology, morphological reconstructions, and diffusion tensor imaging, into a coherent framework. It predicts activity on all scales from the single-neuron spiking activity to the area-level functional connectivity. We compared the model activity with human electrophysiological data and human resting-state functional magnetic resonance imaging (fMRI) data. This comparison reveals that the model can reproduce aspects of both spiking statistics and fMRI correlations if the inter-areal connections are sufficiently strong. Furthermore, we study the propagation of a single-spike perturbation and macroscopic fluctuations through the network. The open-source model serves as an integrative platform for further refinements and future in silico studies of human cortical structure, dynamics, and function.

## Introduction

Brain organization and activity display distinct features across multiple spatial and temporal scales: from the molecular level to whole-brain networks, from sub-millisecond processes to memories that last decades (Deco et al. 2008; Honey et al. 2012; Squire et al. 2015). Impressive technological advancements have made almost all these scales accessible through specialized techniques, which leads to a comprehensive but fragmented picture (Sejnowski et al. 2014). Models have the potential to integrate the diverse data modalities into a unified framework and to bridge across the scales (Pulvermüller et al. 2021). Large-scale, data-driven models at cellular resolution have been constructed for sensory cortex (Reimann et al. 2013; Markram et al. 2015; Girardi-Schappo et al. 2016; Arkhipov et al. 2018; Billeh et al. 2020; Jiang et al. 2024), prefrontal cortex (Hass et al. 2016), hippocampus (Hendrickson et al. 2012; Bezaire et al. 2016; Ecker et al. 2020), cerebellum (Casali et al. 2019; Yamaura et al. 2020), and the olfactory bulb (Migliore et al. 2014, 2015), among others. These models reproduce resting-state activity (e.g. Potjans and Diesmann 2014; Markram et al. 2015; Bezaire et al. 2016; Hass et al. 2016) and stimulus responses (e.g. Arkhipov et al. 2018; Billeh et al. 2020) on various levels of detail. Advances in the simulation technology for large networks of point neurons (Jordan et al. 2018; Einevoll et al. 2019; Pronold et al. 2022a, b) have enabled the step beyond single brain regions to multi-area cortical network models (Schmidt et al. 2018a, b; Lu et al. 2022; see also Izhikevich and Edelman 2008 for a pioneering study).

The multi-area spiking network model of Schmidt et al. (2018b) relates the connectivity of the vision-related areas in one hemisphere of macaque cortex to its dynamics. It integrates cortical architecture and connectivity data, in particular axonal tracing data (Bakker et al. 2012; Markov et al. 2014a, b), into a comprehensive, layer-resolved network of 32 areas. Simulations where the model is poised in a metastable regime just below a transition to a high-activity regime reproduce local and cortico-cortical resting-state activity (Schmidt et al. 2018b): single-cell spiking statistics closely match recordings from macaque V1, and functional connectivity patterns correspond well with macaque functional magnetic resonance imaging (fMRI) data. Moreover, the model yields population bursts that mainly propagate in the feedback direction, akin to empirical findings during visual imagery (Dentico et al. 2014) and in slow-wave sleep (Massimini et al. 2004; Nir et al. 2011; Sheroziya and Timofeev 2014).

In part due to the scarcity of available human data in comparison with other species, only a few large-scale cellularly resolved human brain network models have been built (Izhikevich and Edelman 2008; Lu et al. 2022). The former encompasses a million neurons for most simulations (although a variant with $10^{11}$ neurons was also simulated), while the latter goes up to the full $86$ billion neurons of the human brain. The model of Izhikevich and Edelman (2008) includes thalamocortical interactions and displays self-sustained activity as well as chaotic cortical spiking activity (as observed experimentally by London et al. 2010; but see Priesemann et al. 2014). In contrast, Lu et al. (2022) focus on fMRI data and develop a fitting routine to fine-tune the model in order to reproduce recorded blood-oxygen-level-dependent (BOLD) signals. However, both models neglect cytoarchitectural heterogeneity across areas, for instance using the same average number of incoming synapses per neuron in each brain area. Furthermore, both models simplify laminar patterns of cortico-cortical connectivity, and considerably downscale the number of synapses per neuron. Such downscaling is likely to affect the obtained dynamics, such as the correlation structure of the activity (Van Albada et al. 2015).

Leveraging the increasing availability of human data (e.g. Mohan et al. 2015; Minxha et al. 2020; Berg et al. 2021; Cano-Astorga et al. 2021; Shapson-Coe et al. 2024), we build and simulate a model that encompasses the scales from the single-neuron level to the network of areas in one hemisphere of the human brain with a biological number of neurons and synapses in each local circuit. The model aggregates data across many scales, from electron microscopy data for the density of synapses (DeFelipe et al. 2002b; Cano-Astorga et al. 2021) to whole-brain diffusion tensor imaging (DTI) and fMRI data, supplements it through predictive connectomics (e.g. Barbas and Rempel-Clower 1997; Ercsey-Ravasz et al. 2013; Beul et al. 2017; Hilgetag et al. 2019; Van Albada et al. 2022), and yields activity data on scales from single-neuron spiking activity to area-level correlation patterns.

Simulating large-scale cellularly resolved models requires the efficient use of supercomputers, a thorough understanding of the inherent bottlenecks of these simulations, and state-of-the-art simulation technology. Systematic benchmarking is a significant step toward the optimal use of neuronal simulator technologies such as NEST (Diesmann et al. 2002) on supercomputers (Van et al. 2021; Albers et al. 2022). Furthermore, recent studies have systematically isolated and addressed major contributing factors to long simulation times (Pronold et al. 2022a, b). These optimizations, coupled with a relatively coarse cortical parcellation, limit the simulation times for the model presented here. Shorter simulation times lead to a higher turnover rate of simulations, and enable investigations of more versions and realizations of the model.

First, we describe the data integration into a mesoscale connectome, the detailed construction of the model, and the activity data used to validate the model. We validate the mesoscale connectome against features that were not explicitly built in. Then, we analyze the spiking activity in a version of the model with equal local and inter-areal synaptic strengths, which we call the “base version” of the model. The base version lacks substantial inter-areal interactions, so we systematically increase the inter-areal synaptic weights. Next, we compare the resulting activity with single-neuron spiking statistics and area-level correlation patterns based on fMRI; the “best-fitting version” is achieved when inter-areal synaptic weights are increased relative to local synaptic weights. Finally, we investigate the propagation of both macroscopic fluctuations and single-spike perturbations through the network. These examples illustrate how the model, which we publish as open source, may be used as a basis for a wide range of investigations into human cortical structure, dynamics, and function.

## Materials and methods

### Model construction

In the following text, we detail the composition of the model and the construction of its “mesoconnectome”: the connectivity at the level of neural populations specific to cortical areas and layers. Each of the 34 areas in one hemisphere of the Desikan–Killiany parcellation (Table 1) is modeled as a layer-resolved $1\:\mathrm{mm^{2}}$ microcircuit consisting of leaky integrate-and-fire (LIF) eurons. The layers considered are 2/3, 4, 5, and 6, simplifying laminar subdivisions and ignoring layer 1 in view of its low neuron density. Within each layer, the model distinguishes excitatory and inhibitory neurons. Throughout, we refer to a combination of area, layer, and neural class as a population, for example the population of excitatory neurons in layer 4 of primary visual cortex (area pericalcarine).

**Table 1:** All $34$ areas in the Desikan–Killiany parcellation for one hemisphere with corresponding acronyms.

Long name	Acronym	Long name	Acronym
bankssts	BSTS	parsorbitalis	PORB
caudalanteriorcingulate	CAC	parstriangularis	PTRI
caudalmiddlefrontal	CMF	pericalcarine	PCAL
cuneus	CUN	postcentral	PSTS
entorhinal	ENT	posteriorcingulate	PC
fusiform	FUS	precentral	PREC
inferiorparietal	INFP	precuneus	PCUN
inferiortemporal	IT	rostralanteriorcingulate	RAC
isthmuscingulate	ISTC	rostralmiddlefrontal	RMF
lateraloccipital	LOCC	superiorfrontal	SF
lateralorbitofrontal	LORB	superiorparietal	SP
lingual	LIN	superiortemporal	ST
medialorbitofrontal	MORB	supramarginal	SMAR
middletemporal	MT	frontalpole	FP
parahippocampal	PARH	temporalpole	TP
paracentral	PARC	transversetemporal	TT
parsopercularis	POPE	insula	INS

**Fig. 1:**
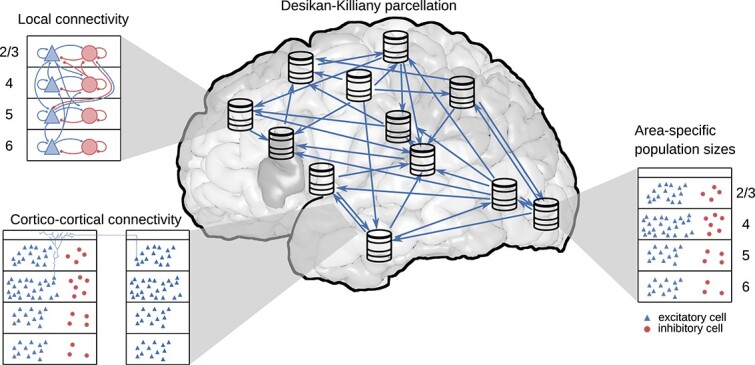
Model overview. The model comprises all $34$ areas of the Desikan–Killiany parcellation (Desikan et al. 2006) in one hemisphere of human cerebral cortex. Each area is modeled by a column with $\mathrm{1\:\mathrm{mm^{2}}}$ cortical surface. Within each column, the full number of neurons and synapses based on anatomical data is included. In total, this leads to $3.47$ million neurons and $42.8$ billion synapses. Both the intrinsic and the inter-areal connectivities are layer- and population-specific.

In each local circuit, the full natural density of neurons and synapses for the modeled layers is used. This leads to a total of $3.47$ million neurons connected via $42.8$ billion model-internal synapses (Fig. 1). The remaining input impinging on the neurons, from non-modeled parts of the brain, is represented as a stochastic drive. The neurons are not assigned spatial coordinates, so that all neurons in a given area, layer, and population are treated as statistically equivalent. The data sources underlying the model construction and validation are listed in Supplementary Table S1 and the heuristics used for the model construction are specified, along with starting points for refinements, in Supplementary Tables S2 and S3. The summary of the model description and model parameters is presented in Tables 2 and 3, respectively.

#### Neuron number

The number of neurons per layer follows from multiplying their volume density $\rho _{\mathrm{neuron}}$ with the layer thickness $h_{\mathrm{layer}}$ and the surface area $A_{\mathrm{column}}$ as $N_{\mathrm{neuron}}=\rho _{\mathrm{neuron}}h_{\mathrm{layer}}A_{\mathrm{column}}$ (here, $A_{\mathrm{column}}=1\:\mathrm{mm^{2}}$). We use the volume density and the layer thickness provided in the seminal work of von Economo and Koskinas (Von and Economo 2009). These data distinguish the layers into finer categories than the ones we use in our model. Therefore, we sum the corresponding “layer thickness overall” and average the corresponding “cell content” values weighted by the relative layer thickness.

Furthermore, the data are provided in the parcellation of von Economo and Koskinas; we use the mapping to the Desikan–Killiany parcellation constructed by Goulas et al. (2016, table 1). In the given mapping, one or more von Economo and Koskinas areas are assigned to each Desikan–Killiany area. For the layer thicknesses, we take the average across the corresponding areas in the parcellation by von Economo and Koskinas (using that the mapping was constructed based on cytoarchitectonic similarity, such that the average is across architectonically similar areas); for the volume densities, we weight the average by the relative thickness of the layers.

To separate the neurons in a given layer into inhibitory and excitatory neurons, we use the layer-resolved relative size of the 2 populations from the electron-microscopy-based reconstruction of cortical tissue in the human temporal lobe by Shapson-Coe et al. (2021, Supplementary Fig. 5B). The resulting fractions of excitatory neurons are 65% in layer 2/3, 79% in layer 4, 78% in layer 5, and 86% in layer 6. The population sizes follow by multiplying the relative population size with the total number of neurons in the layer. The numbers of neurons per population for all areas are listed in Supplementary Table S4.

#### Synapse number

We approximate the volume density of synapses $\rho _{\mathrm{synapse}}=6.6\times 10^{8}\,\mathrm{synapses}/\mathrm{mm}^{3}$ (Cano-Astorga et al. 2021) as constant across cortex (DeFelipe et al. 2002a; Sherwood et al. 2020). This allows us to compute the total number of synapses per area based on their respective volume (Von and Economo 2009), as described above for the number of neurons. The task that remains is to determine the pre- and postsynaptic neurons of these synapses. Once the pre- and postsynaptic populations are determined, the corresponding number of synapses is distributed independently with uniform probability onto the possible connections. In particular, this means that both autapses (connections of a neuron with itself) and multapses (multiple synapses between a pair of neurons) can occur; their occurrence is quantified in Fig. S2.

#### Fraction of local connections

We separate the $N_{\mathrm{synapse}}^{\mathrm{nonlocal}}$ synapses from long-range connections through the white matter from the $N_{\mathrm{synapse}}^{\mathrm{local}}=N_{\mathrm{synapse}}^{\mathrm{total}}-N_{\mathrm{synapse}}^{\mathrm{nonlocal}}$ synapses coming from within the area. To determine the fraction of synapses from long-range projections, we use the scaling rule by Herculano-Houzel et al. (2010):


(1) \begin{align*}& \frac{N_{\mathrm{neuron}}^{\mathrm{nonlocal}}}{N_{\mathrm{neuron}}^{\mathrm{total}}}\propto\frac{1}{\left(N_{\mathrm{neuron}}^{\mathrm{total}}\right)^{0.16}},\end{align*}

i.e. the relative number of neurons sending axons into the white matter decreases with increasing total number of neurons in the gray matter $N_{\mathrm{neuron}}^{\mathrm{total}}$. We determine the proportionality constant using the value $N_{\mathrm{neuron}}^{\mathrm{nonlocal}}/N_{\mathrm{neuron}}^{\mathrm{total}}=0.21$ from tracing data in macaque (Markov et al. 2011,though note that this reflects the intra-hemispheric fraction and neglects inter-hemispheric connections) in combination with $1.4\times 10^{9}$ gray matter neurons in macaque (Collins et al. 2010). With the number of gray matter neurons in human, $N_{\mathrm{neuron}}^{\mathrm{total}}=16\times 10^{9}$ (Herculano-Houzel 2009), we arrive at the estimate $N_{\mathrm{neuron}}^{\mathrm{nonlocal}}/N_{\mathrm{neuron}}^{\mathrm{total}}=0.14$ or $N_{\mathrm{neuron}}^{\mathrm{local}}/N_{\mathrm{neuron}}^{\mathrm{total}}=0.86$ for human cortex.

These numbers determine average fractions of local and non-local synapses in our model; since these synapses are assigned to neuron pairs via random sampling of source and target neurons, the resulting fractions of sending neurons can actually differ from the given percentages. Further, the non-local connectivity is made area-specific according to DTI data as specified in the section “*Long-Range Projections.*” We assume that the fraction of neurons sending axons into the white matter equals the fraction of synapses from long-range projections, i.e. from inter-areal cortico-cortical and subcortical sources; in particular, all connections between different cortical areas are treated as white-matter connections.

#### Local connectivity

The $N_{\mathrm{synapse}}^{\mathrm{local}}$ local synapses need a further distinction: $N_{\mathrm{synapse}}^{\mathrm{internal}}$ synapses where the presynaptic neuron is part of the simulated column and $N_{\mathrm{synapse}}^{\mathrm{external}}$ synapses where the presynaptic neuron is outside of the simulated column, i.e. in the remainder of the area. To split these 2 categories, we use the spatial connection probability $p(\boldsymbol{x}_{1}\,|\,\boldsymbol{x}_{2})$ between a neuron located at $\boldsymbol{x}_{1}$ and another neuron at $\boldsymbol{x}_{2}$, which we assume to be a spatially homogeneous 3-dimensional exponential distribution $p(\boldsymbol{x}_{1}\,|\,\boldsymbol{x}_{2})\propto \exp (-|\boldsymbol{x}_{1}-\boldsymbol{x}_{2}|/\lambda _{\mathrm{conn}})$ with decay constant $\lambda _{\mathrm{conn}}=160\,\mu \mathrm{m}$ (Packer and Yuste 2011; Perin et al. 2011). From $p(\boldsymbol{x}_{1},\boldsymbol{x}_{2})=p(\boldsymbol{x}_{1}\,|\,\boldsymbol{x}_{2})p(\boldsymbol{x}_{2})$, where $p(\boldsymbol{x}_{2})$ is assumed to be constant reflecting a uniform distribution of neurons across space, we obtain the connection density $\rho _{\mathrm{internal}}$ within the column as


(2) \begin{align*} \rho_{\mathrm{internal}} & \propto\int_{\mathrm{col.}}d\boldsymbol{x}_{1}\,\int_{\mathrm{col.}}d\boldsymbol{x}_{2}\,\exp(-|\boldsymbol{x}_{1}-\boldsymbol{x}_{2}|/\lambda_{\mathrm{conn}}),\end{align*}

where the proportionality factor is the normalization constant of $p(\boldsymbol{x}_{1},\boldsymbol{x}_{2})$. We calculate the connection density assuming cylindrical columns. In cylindrical coordinates, using $d\boldsymbol{x}=rdrd\phi dz$ and $\int _{0}^{a}dx_{1}\,\int _{0}^{a}dx_{2}\,f(|x_{2}-x_{1}|)=2\int _{0}^{a}dy\,(a-y)f(|y|)$ simplifies this integral to


(3) \begin{align*} \rho_{\mathrm{internal}}\propto & 4\int_{0}^{r_{\mathrm{col.}}}dr_{1}\,\int_{0}^{r_{\mathrm{col.}}}dr_{2}\,r_{1}r_{2}\nonumber \\ & \times\int_{0}^{2\pi}d\phi\,(2\pi-\phi)\int_{0}^{h}dz\,(h-z)\nonumber \\ & \times\exp(-d(r_{1},r_{2},\phi,z)/\lambda_{\mathrm{conn}}),\end{align*}

with $d(r_{1},r_{2},\phi ,z)=\sqrt{r_{1}^{2}-2r_{1}r_{2}\cos \phi +r_{2}^{2}+z^{2}}$, the radius of the column $r_{\mathrm{col.}}$, and the total height of the column $h$. For the connection density $\rho _{\mathrm{external}}$ that the postsynaptic neuron is in the column but the presynaptic neuron outside of it, the domain outside of the column has to be integrated: $\int _{\mathrm{col.}}d\boldsymbol{x}_{1}\to \int _{\neg \mathrm{col.}}d\boldsymbol{x}_{1}$. Approximating the entire area as a cylinder, this leads to the replacement $\int _{0}^{r_{\mathrm{col.}}}dr_{1}\to \int _{r_{\mathrm{column}}}^{r_{\mathrm{area}}}dr_{1}$, where $r_{\mathrm{area}}$ is the radius of the larger cylinder, i.e.


(4) \begin{align*} \rho_{\mathrm{external}}\propto & 4\int_{r_{\mathrm{col.}}}^{r_{\mathrm{area}}}dr_{1}\,\int_{0}^{r_{\mathrm{col.}}}dr_{2}\,r_{1}r_{2}\nonumber \\ & \times\int_{0}^{2\pi}d\phi\,(2\pi-\phi)\int_{0}^{h}dz\,(h-z)\nonumber \\ & \times\exp(-d(r_{1},r_{2},\phi,z)/\lambda_{\mathrm{conn}}),\end{align*}

with the same normalization factor as for the internal synapses. Here, radius is approximated as $r_{\mathrm{area}}\approx \sqrt{A_{\mathrm{area}}/\pi }$ based on the surface area $A_{\mathrm{area}}$. The remaining integrals are solved numerically using the adaptive multidimensional quadrature implemented in SciPy (Virtanen et al. 2020). $\rho _{\mathrm{internal}}$ and $\rho _{\mathrm{external}}$ are used to determine the number of synapses with neurons within and outside of the column, respectively:


(5) \begin{align*} N_{\mathrm{synapse}}^{\mathrm{internal}} & =\frac{\rho_{\mathrm{internal}}}{\rho_{\mathrm{internal}}+\rho_{\mathrm{external}}}N_{\mathrm{\mathrm{synapse}}}^{\mathrm{local}}, \end{align*}

(6) \begin{align*} N_{\mathrm{\mathrm{synapse}}}^{\mathrm{external}} & =\frac{\rho_{\mathrm{external}}}{\rho_{\mathrm{internal}}+\rho_{\mathrm{external}}}N_{\mathrm{\mathrm{synapse}}}^{\mathrm{local}}. \end{align*}

Note that although we keep $r_{\mathrm{col.}}$ the same for all areas, both $\rho _{\mathrm{internal}}$ and $\rho _{\mathrm{external}}$ are area-specific because their thickness $h$, the total surface area, and the neuron densities vary.

For the local connectivity within the column, comprising $N_{\mathrm{synapse}}^{\mathrm{internal}}$ synapses, we use the model of Potjans and Diesmann (2014) as a blueprint. More precisely, we use the average number of synapses $q_{\mathrm{B}\to \mathrm{A}}^{\mathrm{PD}}$ between a neuron in source population $\mathrm{B}$ and a neuron in target population $\mathrm{A}$. We combine these average numbers of synapses with the number of neurons $N_{\mathrm{neuron}}^{\mathrm{B}}$, $N_{\mathrm{neuron}}^{\mathrm{A}}$ in the pre- and postsynaptic population:


(7) \begin{align*} N_{\mathrm{synapse}}^{\mathrm{B}\to\mathrm{A}} & =\frac{N_{\mathrm{neuron}}^{\mathrm{B}}q_{\mathrm{B}\to\mathrm{A}}^{\mathrm{PD}}N_{\mathrm{neuron}}^{\mathrm{A}}}{\sum_{\mathrm{A},\mathrm{B}}N_{\mathrm{neuron}}^{\mathrm{B}}q_{\mathrm{B}\to\mathrm{A}}^{\mathrm{PD}}N_{\mathrm{neuron}}^{\mathrm{A}}}N_{\mathrm{synapse}}^{\mathrm{internal}}.\end{align*}

Equation (7) keeps the relative average number of synapses per pair of neurons (i.e. relative to the other population pairs) equal to the respective value in Potjans and Diesmann (2014) by construction. In particular, for agranular areas, Eq. (7) assigns no synapses to layer 4 while preserving the anatomically determined number of synapses. The resulting average numbers of model-internal local synapses per neuron are listed for each target population in Supplementary Table S5.

The $N_{\mathrm{synapse}}^{\mathrm{external}}$ synapses from outside the column are also distributed based on Potjans and Diesmann (2014). Here, we use the indegrees $K_{\mathrm{ext}\to \mathrm{A}}^{\mathrm{PD}}$ ($k_{\mathrm{ext}}$(reference) in their table 5) and the number of neurons in the postsynaptic population $N_{\mathrm{neuron}}^{\mathrm{A}}$ to scale the number of synapses:


(8) \begin{align*} N_{\mathrm{synapse}}^{\mathrm{ext}\to\mathrm{A}} & =\frac{K_{\mathrm{ext}\to\mathrm{A}}^{\mathrm{PD}}N_{\mathrm{neuron}}^{\mathrm{A}}}{\sum_{\mathrm{A}}K_{\mathrm{ext}\to\mathrm{A}}^{\mathrm{PD}}N_{\mathrm{neuron}}^{\mathrm{A}}}N_{\mathrm{synapse}}^{\mathrm{external}}.\end{align*}

Thus, the external indegrees from Potjans and Diesmann (2014) determine the relative external indegrees for the different populations but not their absolute values. In both Eq. (7) and Eq. (8), we round the final result to obtain an integer number of synapses. The resulting external indegrees are not explicitly represented in the model by simulated neurons. Rather, they are simplified as excitatory external inputs, as described in the next sections.

#### Long-range projections

The $N_{\mathrm{synapse}}^{\mathrm{nonlocal}}$ synapses could belong to intra- or inter-hemispheric inter-areal projections, or to projections from subcortical structures. Retrograde tracing in macaque showed that only about $5\%$ of the presynaptic neurons are located in nonadjacent cortical areas within the hemisphere and only about $1\%$ are located in subcortical structures (Markov et al. 2011). Furthermore, contralateral projections (from the other hemisphere) tend to form only a small fraction of the combined inter-areal projections (e.g. Dehay et al. 1988; Barbas et al. 2005; Rosen and Halgren 2022), although this fraction is regionally specific (Ruddy et al. 2017). Based on these observations and the assumption that the fraction of presynaptic neurons equals the fraction of the corresponding synapses, we neglect both subcortical and inter-hemispheric projections, i.e. we treat all $N_{\mathrm{synapse}}^{\mathrm{nonlocal}}$ synapses as belonging to intra-hemispheric inter-areal projections. Furthermore, we assume that the presynaptic neurons are inside the simulated column in the respective presynaptic area. Thus, we do not consider spatial divergence or convergence of connections beyond the $1\:\mathrm{mm^{2}}$ scale.

We define the area-level connectivity according to processed DTI data from Goulas et al. (2016), which is based on data from the Human Connectome Project (Van Essen et al. 2013). For a given target area $X$, we distribute the synapses among the source areas based on the relative number of streamlines $\mathrm{NoS}_{\mathrm{Y}\to \mathrm{X}}$ in the DTI data:


(9) \begin{align*} N_{\mathrm{synapse}}^{\mathrm{Y}\to\mathrm{X}} & =\frac{\mathrm{NoS}_{\mathrm{Y}\to\mathrm{X}}}{\sum_{\mathrm{Z}}\mathrm{NoS}_{\mathrm{Z}\to\mathrm{X}}}N_{\mathrm{synapse}}^{\mathrm{nonlocal}}.\end{align*}

As before, we round the resulting value.

A comprehensive dataset on the layer specificity of the presynaptic neurons based on retrograde tracing is available for macaque (Markov et al. 2014a, b). Not only in this species but also in cat, the layer specificity as measured by the fraction of supragranular labeled neurons $\mathrm{SLN}$ is systematically related to the cytoarchitecture (Van Albada et al. 2022). For our human model, we assume the same quantitative relationship as in macaque, for lack of the relevant human-specific data. Fitting a beta-binomial model with a probit link function to the macaque data yields (Schmidt et al. 2018a)


(10) \begin{align*} \mathrm{SLN}(\mathrm{B} & \to\mathrm{A})=\Phi\left(a_{0}+a_{1}\log(\rho_{\mathrm{neuron}}^{\mathrm{A}}/\rho_{\mathrm{neuron}}^{\mathrm{B}})\right),\end{align*}

where $\Phi (x)=\frac{1}{2} [1+\mathrm{erf} (x/\sqrt{2}) ]$ denotes the cumulative distribution function of the standard normal distribution and the fitted parameters are $a_{0}=-0.152$ and $a_{1}=-1.534$. We use the human neuron densities in Eq. (10) to estimate the laminar origin in human. The $\mathrm{SLN}$ value allows determining whether the origin is in layer $2/3$ or not. Excluding layer $4$, which does not form long-range projections (Markov et al. 2014b), the 2 infragranular layers $5$ and $6$ still need to be distinguished. To this end, we simply use the relative size of the 2 populations to distribute the remaining synapses.

On the target side, anterograde tracing can specify the layer specificity. However, there are no comprehensive datasets of anterograde tracing in non-human primates available to date. Hence, we use the collected data from the CoCoMac database (Stephan et al. 2001), which aggregates data across many tracing studies. Relating the target patterns from anterograde tracing to the $\mathrm{SLN}$ value reveals 3 categories of target patterns (Schmidt et al. 2018a):


\begin{align*} \mathrm{SLN}>65\%: & [4]\\ 35\%\le\mathrm{SLN}\le65\%: & [1,2/3,4,5,6]\\ \mathrm{SLN}<35\%: & [1,2/3,5,6], \end{align*}

where layer $4$ is replaced by $2/3$ in the first case for agranular target areas (Beul and Hilgetag 2015). Using the $\mathrm{SLN}$ value to distinguish feedforward ($\mathrm{SLN}>65\%$), lateral ($35\%\le \mathrm{SLN}\le 65\%$), and feedback ($\mathrm{SLN}<35\%$) connections, this implies that feedforward connections target layer $4$, feedback connections avoid layer $4$, and lateral connections show no distinct pattern. For the quantitative distribution of the synapses onto the layers included in the respective target pattern, we use the relative thickness of the layer in relation to all layers of the target pattern.

Thus far, we determined the location of the synapse in the target layer. Next, we decide whether the postsynaptic neuron of a synapse in a given layer is excitatory or inhibitory based on the analysis of the data by Binzegger et al. (2004) in Schmidt et al. (2018a, Table S11). To this end, we sum the target probabilities for postsynaptic neurons across all layers separately for excitatory and inhibitory neurons. This yields the probability for a synapse in a given layer to have an excitatory or inhibitory postsynaptic neuron in any layer. However, we take one exception into account: for feedback connections ($\mathrm{SLN}<35\%$), we fix the fraction of excitatory target cells to $93\%$ (Schmidt et al. 2018a) because feedback connections have been found to preferentially target excitatory neurons (Johnson and Burkhalter 1996; Anderson et al. 2011).

To finally determine the postsynaptic neuron, we assume that all inhibitory postsynaptic neurons are in the same layer as the synapse. For the excitatory neurons, we take the dendritic morphology into account. Using morphological reconstructions of human pyramidal cells in temporal cortex (Mohan et al. 2015) (for a subset of the data see Mohan et al. 2023), we calculate the layer-resolved length of dendrites for neurons with the soma in a given layer. Assuming a constant density of synapses along the dendrites, the ratio of the length $\ell _{\mathrm{A},\mathrm{B}}$ of dendrites in layer $\mathrm{A}\in [1,2/3,4,5,6]$ belonging to neurons with soma in layer $\mathrm{B}\in [2/3,4,5,6]$ to the total length of dendrites in this layer, $\sum _{\mathrm{B}}\ell _{\mathrm{A},\mathrm{B}}$, determines the probability that the postsynaptic cell is in layer $\mathrm{B}$ given that the synapse is in layer $\mathrm{A}$: $P(\mathrm{soma\,in\,B}\,|\,\mathrm{synapse\,in\,A})=\ell _{\mathrm{A},\mathrm{B}}/\sum _{\mathrm{B}}\ell _{\mathrm{A},\mathrm{B}}$.

Ultimately, we only need the location of the postsynaptic neuron but not the location of the synapse. Thus, we multiply $P(\mathrm{soma\,in\,B}\,|\,\mathrm{synapse\,in\,A})$ with the distribution of the synapses across the layers and marginalize the synapse location. The average numbers of incoming long-range synapses per neuron for all areas in our model are listed in Supplementary Table S6.

#### Further model specifications

##### Neuron parameters

We use the LIF neuron model with exponential postsynaptic currents (Gerstner et al. 2014) for all neurons. To determine the parameter values, we analyzed the LIF models from the Allen Cell Types Database (https://celltypes.brain-map.org/; Teeter et al. 2018; Berg et al. 2021) which were fitted to human neurons. For both excitatory and inhibitory cells, we fix the leak and reset potential to $\mathrm{V}_{\mathrm{L}}=\mathrm{V}_{\mathrm{reset}}=-70\,\mathrm{mV}$. For the threshold potential $\mathrm{V}_{\mathrm{th}}$, the membrane time constant $\mathrm{\tau }_{\mathrm{m}}$, and the membrane capacitance $\mathrm{C}_{\mathrm{m}}$, we fitted a log-normal distribution using maximum likelihood estimation to the distribution of the respective parameter for all cells in which the LIF model had an explained variance above $0.75$ to ensure a good fit of the LIF model. For convenience, we parameterize the log-normal distribution using the mean and the coefficient of variation $\mathrm{CV}$. The resulting mean threshold potential is $\mathrm{V}_{\mathrm{th}}=-45\,\mathrm{mV}$ for both excitatory and inhibitory cells with $\mathrm{CV}=0.21$ and $\mathrm{CV}=0.22$ for excitatory and inhibitory cells, respectively. The resulting mean capacitance is $\mathrm{C}_{\mathrm{m}}=220\,\mathrm{pF}$ and $\mathrm{C}_{\mathrm{m}}=100\,\mathrm{pF}$ with $\mathrm{CV}=0.22$ and $\mathrm{CV}=0.34$ for excitatory and inhibitory cells, respectively. To account for the high-conductance state in vivo (Destexhe et al. 2003), we lower the membrane time constant to $\mathrm{\tau }_{\mathrm{m}}=10\,\mathrm{ms}$ on average with $\mathrm{CV}=0.55$ and $\mathrm{CV}=0.43$ for excitatory and inhibitory cells, respectively. We do not distribute the synaptic time constants, which we fix to $\mathrm{\tau }_{\mathrm{s}}=2\,\mathrm{ms}$, and the absolute refractory period of $\mathrm{t}_{\mathrm{ref}}=2\,\mathrm{ms}$.

In all simulations shown in the main text, the neuron parameters are not distributed, i.e. all coefficients of variation were set to $\mathrm{CV}=0$. Simulations with distributed neuron parameters are shown in the appendix.

##### Synapse parameters

We use static synapses with a transmission probability of $100\,\%$. Excitatory postsynaptic potentials follow a truncated normal distribution with average amplitude $0.1\,\mathrm{mV}$ and relative standard deviation of $10\,\%$. The inhibitory postsynaptic potentials also follow a truncated normal distribution with a factor $g=5$ larger absolute value of the mean and standard deviation. Excitatory (inhibitory) weights are truncated below (above) zero; values outside of the allowed range are redrawn.

Postsynaptic potentials are converted into postsynaptic currents using the conversion factor


(11) \begin{align*}& \frac{\mathrm{PSC}}{\mathrm{PSP}}=\frac{C_{\mathrm{m}}}{\mathrm{\tau}_{\mathrm{m}}}\epsilon^{-1/(1-\epsilon)},\qquad\epsilon=\frac{\mathrm{\tau}_{\mathrm{s}}}{\mathrm{\tau}_{\mathrm{m}}}.\end{align*}

Note that the conversion factor depends on both the synapse parameters ($\mathrm{\tau }_{\mathrm{s}}$) and the postsynaptic neuron parameters ($\mathrm{\tau }_{\mathrm{m}}$, $C_{\mathrm{m}}$).

We introduce several scaling factors that affect the postsynaptic potentials: first, the synaptic weights of the synapses within a column from layer 4 excitatory neurons to layer 2/3 excitatory neurons are increased 2-fold, in agreement with the blueprint (Potjans and Diesmann 2014). Second, we introduce a scaling factor $\chi _{I}$ for the cortico-cortical synapses targeting inhibitory neurons. This scaling factor stabilizes the column with respect to inter-areal input. For all simulations shown in the main text, it is set to $2.0$. Third, we introduce a scaling factor $\chi $ for the inter-areal connections onto both excitatory and inhibitory neurons. Increasing this factor leads to the best-fitting version Figs. 5 and 6. For inter-areal synapses onto inhibitory neurons, $\chi _{I}$ and $\chi $ are multiplied with each other.

##### Delays

Within a column, the average transmission delay is $1.5\,\mathrm{ms}$ for excitatory and $0.75\,\mathrm{ms}$ for inhibitory connections. For the inter-areal connections, we assume an average conduction velocity of $3.5\,\mathrm{m/s}$ (Girard et al. 2001). Dividing the fiber length between 2 areas, obtained through tractography (Goulas et al. 2016), by this conduction velocity, we obtain the average delay between the 2 areas. All delays follow a truncated log-normal distribution with a relative standard deviation of $50\,\%$. Delays are truncated below the resolution of the simulation; values outside of the allowed range are redrawn.

##### External input

We determined the number of synapses from non-simulated presynaptic neurons in Eq. (8). The postsynaptic potentials follow a truncated normal distribution with average $\mathrm{w}_{\mathrm{ext}}=0.1\,\mathrm{mV}$ and relative standard deviation of $10\,\%$. Note that, for simplicity, we assume that the external input is exclusively excitatory. We keep the mean input, measured relative to rheobase, fixed at $\eta _{\mathrm{ext}}=1.1$ and determine the rate of the driving Poisson processes by


(12) \begin{align*} \nu_{\mathrm{ext}}^{\mathrm{A}} & =\frac{\mathrm{V}_{\mathrm{th}}-\mathrm{V}_{\mathrm{L}}}{\mathrm{\tau}_{\mathrm{m}}\mathrm{w}_{\mathrm{ext}}K_{\mathrm{A}}^{\mathrm{ext}}}\eta_{\mathrm{ext}},\end{align*}

with $K_{\mathrm{A}}^{\mathrm{ext}}=N_{\mathrm{synapse}}^{\mathrm{ext}\to \mathrm{A}}/N_{\mathrm{neuron}}^{\mathrm{A}}$ (extrinsic indegrees for each population listed in Supplementary Table S7). We further introduce 2 scaling factors for the postsynaptic potentials arriving at excitatory neurons in layers $5$ and $6$, respectively. For all simulations shown, the first scaling factor is set to $1.05$ and the second to $1.15$. The resulting $\nu _{\mathrm{ext}}$, for our parameter set, spans a range from $0$ to $13.35\,\mathrm{spikes}/\mathrm{s}$, with a mean of $3.59\pm 2.03\,\mathrm{spikes}/\mathrm{s}$. An isolated neuron receiving only the external input fires in the range of $35.0$ to $50.0\,\mathrm{spikes}/\mathrm{s}$.

**Table 2:** Model description after Nordlie et al. (2009).

**Model summary**	
Populations	34 areas (Table 1) with a total of 254 populations. The model consists of about 3.5 million neurons and 43 billion synapses.
Geometry	—
Connectivity	area- and population-specific but otherwise random
Neuron model	LIF, fixed absolute refractory period (voltage clamp)
Synapse model	exponential postsynaptic currents
Plasticity	—
Input	independent homogeneous Poisson spike trains
Measurements	spiking activity
**Populations**	
Type	Cortex
Elements	LIF neurons
Number of populations	34 areas with 8 populations each (areas caudalanteriorcingulate, caudalmiddlefrontal, entorhinal, lateraloccipital, parsorbitalis, precentral, rostralanteriorcingulate have 6, and the parahippocampal area has 4), 2 per layer
Population size	$N$ (area- and population-specific)
**Connectivity**	
Type	source and target neurons drawn randomly with replacement (allowing autapses and multapses) with area- and population-specific connection probabilities. The total number of synapses between populations is fixed, corresponding to the “Random, fixed total number” rule described by Senk et al. (2022).
Weights	fixed, drawn from normal distribution with mean $J$ such that postsynaptic potentials have a mean amplitude of $0.1\:\mathrm{mV}$ and standard deviation $\delta J = 0.1 J$; 4E to 2/3E increased by factor $2$ (cf. Potjans and Diesmann 2014); weights of inhibitory connections increased by factor $g$; excitatory weights $<0$ and inhibitory weights $>0$ are redrawn; inter-areal weights onto inhibitory populations increased by factor $\chi _{I}$ and onto excitatory and inhibitory populations increased by factor $\chi $
Delays	fixed, drawn from truncated lognormal distribution with mean $d$ and standard deviation $\delta d = 0.5 d$; delays of inhibitory connections factor $2$ smaller; delays rounded to the nearest multiple of the simulation step size $h=0.1\,\mathrm{ms}$, inter-area delays drawn from a truncated lognormal distribution with mean $d=s/v_{\mathrm{t}}$, with distance $s$ and average transmission speed $v_{\mathrm{t}}=3.5\,\mathrm{m/s}$ (Girard et al. 2001); and standard deviation $\delta d=d/2$, distances determined as the median of the distances between all vertex pairs of the 2 areas in the DTI data (Goulas et al. 2016), delays $<0.1\,\mathrm{ms}$ before rounding are redrawn
**Neuron and synapse model**	
Name	LIF neuron
Type	LIF, exponential synaptic current inputs
Subthreshold dynamics	$\frac{dV}{dt}$ $= -\frac{V-E_{\mathrm{L}}}{\tau _{\mathrm{m}}} + \frac{I_{\mathrm{s}}(t)}{C_{\mathrm{m}}}$ $\text{if} (t> t^{*}+\tau _{\mathrm{r}})$, $V(t)$ $= V_{\mathrm{r}}$ else, $I_{\mathrm{s}}(t)=\sum _{i,k}J_{k}\,e^{-(t-t_{i}^{k})/\tau _{\mathrm{s}}}\Theta (t-t_{i}^{k})$, $k$: neuron index, $i$: spike index, $\Theta $: Heaviside step function
Spiking	If $V(t-)<\theta \wedge V(t+)\geq \theta $ 1. set $t^{*}=t$, 2. emit spike with time stamp $t^{*}$
**Input**	
Type	Background
Target	LIF neurons
Description	Independent homogeneous Poisson spike trains to all neurons in the network; rate fixed such that the mean input, measured relative to rheobase, is $\eta _{\mathrm{ext}}=1.1$
**Measurements**	
Spiking activity	

**Table 3:** Parameter specification for synapses and neurons.

**Name**	**Value**	**Description**
**Synapse parameters**		
$J\pm \delta J$	Intra-areal connections: $16.4\pm 1.6\mathrm{\:pA}$ onto excitatory and $7.5\pm 0.8\mathrm{\:pA}$ onto inhibitory neurons prior to the application of scaling factors, inter-areal connections scaled as $J_{\mathrm{cc}}=\chi J$ onto excitatory and $J_{\mathrm{cc}}=\chi \chi _{I}J$ onto inhibitory neurons	excitatory synaptic strength
$g$	$g=5$	relative inhibitory synaptic strength
$d_{\mathrm{e}}\pm \delta d_{\mathrm{e}}$	$1.5\pm 0.75\:\mathrm{ms}$	local excitatory transmission delay
$d_{\mathrm{i}}\pm \delta d_{\mathrm{i}}$	$0.75\pm 0.375\:\mathrm{ms}$	local inhibitory transmission delay
$d\pm \delta d$	$d=s/v_{\mathrm{t}}\pm \frac{1}{2}s/v_{\mathrm{t}}$	inter-area transmission delay, with $s$ the distance between areas
$v_{\mathrm{t}}$	$3.5\,\mathrm{m/s}$	transmission speed
**Neuron parameters**		
$\tau _{\mathrm{m}}$	$10\:\mathrm{ms}$	membrane time constant
$\tau _{\mathrm{r}}$	$2\:\mathrm{ms}$	absolute refractory period
$\tau _{\mathrm{s}}$	$2\:\mathrm{ms}$	postsynaptic current time constant
$C_{\mathrm{m}}$	$220\:\mathrm{pF}$ for excitatory neurons,$100\:\mathrm{pF}$ for inhibitory neurons	membrane capacity
$V_{\mathrm{r}}$	$-70\:\mathrm{mV}$	reset potential
$\theta $	$-45\:\mathrm{mV}$	fixed firing threshold
$E_{\mathrm{L}}$	$-70\:\mathrm{mV}$	leak potential

### Activity data

#### Experimental spiking data

Minxha et al. (2020) recorded data from 13 adult epilepsy patients under evaluation for surgical treatment using depth electrodes in medial frontal cortex. In total, they recorded $767$ neurons within $320$ trials and extracted spikes using a semi-automated spike sorting algorithm. For our analysis, we disregard task-related activity and use only the 2 s of activity that were recorded before stimulus onset. The data are publicly available via the Open Science Framework at http://doi.org/10.17605/OSF.IO/U3KCP.

#### Temporal hierarchy from model spiking data

To study the propagation of macroscopic fluctuations through the network, we determine the dominant order of activations of the areas, which we term “temporal hierarchy,” in the best-fitting version of the model. Spike trains from simulations of 10 s biological time (after an initial 2.5 s that are discarded) using the best-fitting parameters were converted to spike rate signals by aggregating across layers and 1-ms time intervals. For each pair of areas, delay times (positive or negative) were estimated as the peak location of the cross-correlation function between their spike rate signals. When multiple peaks of similar height were detected, the delay was selected based on specific criteria: if the corresponding delays had the same sign, the one closest to zero was selected. Otherwise, the case was labeled “undecided.” To further refine the delay estimates, the time series was divided into 9 segments to get 9 independent estimates of the delay. When the median absolute deviation of these 9 peaks was more than 3 ms, the previously computed delay was rejected. The resulting data formed a matrix consisting of delay times between pairs of areas, along with “undecided” labels for ambiguous cases. The method described by Schmidt et al. (2018b) was used to minimize the delays predicted from the hierarchy (starting with the most leading and ending with the most lagging area) and the actual delay estimates. This hierarchy thus represents the main direction of activity flow across the areas, apart from oscillatory activity that we largely discard because of the ambiguous directionality it implies.

#### FMRI data

##### Participants

MRI data were obtained from 19 participants (7 female, age range = 21 to 33 years, mean age = 25 years) with normal or corrected-to-normal visual acuity. All participants provided written informed consent after receiving full information about experimental procedures and were compensated for participation through either monetary reward or course credit. All procedures were conducted with approval from the local Ethical Committee of the Faculty of Psychology and Neuroscience at Maastricht University.

##### Magnetic resonance imaging

Anatomical and functional images were acquired at Maastricht Brain Imaging Centre (Maastricht University) on a whole-body Magnetom 7T research scanner (Siemens Healthineers, Erlangen, Germany) using a 32-channel head-coil (Nova Medical Inc.; Wilmington, MA, USA). Anatomical data were collected prior to functional data with an MP2RAGE (Marques et al. 2010) imaging sequence [$240$ slices, matrix = $320\times 320$, voxel size = $0.65\times 0.65\times 0.65$ mm^3^, first inversion time (TI1) = 900 ms, second inversion time (TI2) = 2750 ms, echo time (TE) = 2.51 ms, repetition time (TR) = 5000 ms, first nominal flip angle = 5$^{\circ }$, and second nominal flip angle = 3$^{\circ }$, GRAPPA = $2$]. Functional images were acquired using a gradient-echo echo-planar (Moeller et al. 2010) imaging sequence ($84$ slices, matrix = $186\times 186$, voxel size = $1,6\times 1.6\times 1.6$ mm^3^, TE = 22 ms, TR = 1500 ms, nominal flip angle = 63$^{\circ }$, GRAPPA = $2$, multi-band factor = $4$). In addition, after the first functional run, we recorded 5 functional volumes with opposed phase encoding directions to correct for EPI distortions that occur at higher field strengths (Andersson et al. 2003).

Participants underwent 5 functional runs comprising a resting-state measurement, 3 individual task measurements, and a task-switching paradigm wherein participants repeatedly performed each of the 3 tasks. With the exception of the task-switching run, which lasted 9.5 min, all functional runs lasted 15 min. Since task-related runs were not included in this study, they will not be discussed further. However, it is noteworthy that resting-state runs always preceded task-related runs to prevent carry-over effects (Grigg and Grady 2010). Participants were instructed to close their eyes during resting-state runs and otherwise to let their mind wander freely.

##### Processing of (f)MRI data

Anatomical images were downsampled to $0.8\times 0.8\times 0.8$ mm^3^ and subsequently automatically processed with the longitudinal stream in FreeSurfer (http://surfer.nmr.mgh.harvard.edu/) including probabilistic atlas-based cortical parcellation according to the Desikan–Killiany (DK) atlas (Desikan et al. 2006). Initial preprocessing of functional data was performed in BrainVoyager 20 (version 20.0; Brain Innovation; Maastricht, The Netherlands) and included slice scan time correction and (rigid body) motion correction wherein all functional runs were aligned to the first volume of the first functional run. EPI distortions were then corrected using the COPE (Correction based on Opposite Phase Encoding) plugin of BrainVoyager that implements a method similar to that described in Andersson et al. (2003) and the “topup” tool implemented in FSL (Smith et al. 2004). The pairs of reversed phase encoding images recorded in the beginning of the scanning session were used to estimate the susceptibility-induced off-resonance field and correct the distortions in the remaining functional runs. This was followed by wavelet despiking (Patel and Bullmore 2016) using the BrainWavelet Toolbox (brainwavelet.org) for MATLAB (2019a, The MathWorks, Natick, MA). Subsequently, high-pass filtering was performed in BrainVoyager with a frequency cutoff of 0.01Hz and to register functional images to participants’ anatomical images. Using MATLAB, functional data were then cleaned further by regressing out a global noise signal given by the first 5 principal components of signals observed within the cerebrospinal fluid of the ventricles (Behzadi et al. 2007). Finally, voxels were uniquely assigned to one of $68$ cortical regions of interest (ROIs) and an average BOLD signal for each ROI was obtained as the mean of the time-series of its constituent voxels.

### Code and workflow

The entire workflow of the model, from data preprocessing to simulation and the final analysis, relies on the Python programming language (https://www.python.org/) version $3.9$ in combination with NumPy (Harris et al. 2020) version $1.21.3$, SciPy (Virtanen et al. 2020) version $1.7.1$, pandas (McKinney 2010) version $1.3.4$, Matplotlib (Hunter 2007) version $3.4.3$, networkx version $2.4$ (Hagberg et al. 2008), and seaborn (Waskom 2021) version $0.11.2$. All simulations were performed using the NEST simulator (Gewaltig and Diesmann 2007) version $2.20.2$ (Fardet et al. 2021) on the JURECA-DC supercomputer. A simulation of $10\:\mathrm{s}$ biological time takes approximately $200$ core-hours ($1\:\mathrm{min}$ build phase + $15\:\mathrm{min}$ for $10\:\mathrm{s}$ biological time on $768$ cores). The workflow is structured using Snakemake (Köster and Rahmann 2012). For the mean-field analysis, we used the NNMT toolbox (Layer et al. 2022).

## Results

### Human mesoscale connectome

The model comprises all $34$ areas of one hemisphere of human cortex in the Desikan–Killiany parcellation (Desikan et al. 2006). Each area is modeled by a $1\:\mathrm{mm^{2}}$ column and the columns are connected through long-range projections (see Fig. 1). We here give a brief summary of the model construction complementing the details in the Materials and methods.

We distinguish 2 classes of neurons, excitatory and inhibitory, and account for the layered structure of cortex. At this level of modeling, the connectivity statistics between neurons in both classes and all layers are needed, which are not straightforwardly delivered by current experimental techniques. Accordingly, we combine available data with predictive connectomics to arrive at a human mesoconnectome at a layer- and population-resolved level. The lack of data on the connectivity is the main reason for considering only 2 classes of neurons. While a recent study defines $45$ inhibitory and $24$ excitatory neuron types in human (Hodge et al. 2019), including this diversity would require a huge number of cell-type-specific connection probabilities. This is not yet feasible because no connectivity data are available at such a fine granularity; hence, we restrict the model to 2 classes of neurons, as done in earlier studies (Potjans and Diesmann 2014; Schmidt et al. 2018a, b).

#### Mesoscale connectome

**Fig. 2:**
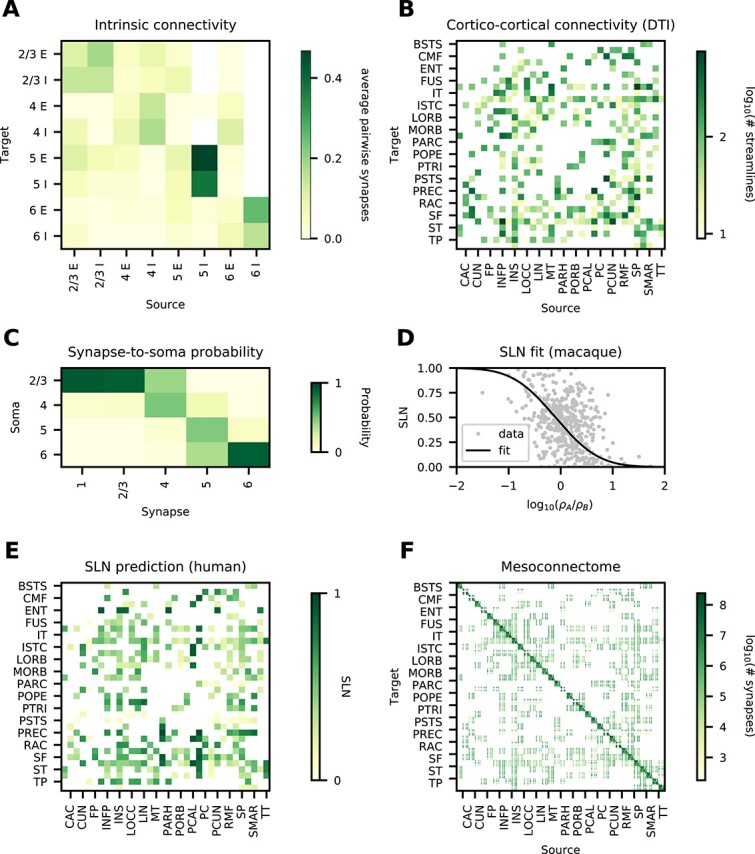
Data and predictive connectomics. (**A**) Within-area connectivity blueprint (average number of synapses per pair of neurons). (**B**) Inter-areal connectivity based on DTI (number of streamlines); see Table 1 for acronyms. (**C**) Probability for inter-areal synapses in a given layer to be established on neurons with cell body in a given layer, estimated from human neuron morphologies. (**D**) Relation of neuron densities of source area *B* and target area *A* with laminar source pattern (fraction of supragranular labeled neurons, $\mathrm{SLN}$) in macaque. (**E**) Predicted source pattern ($\mathrm{SLN}$) in human. (**F**) Layer- and population-resolved mesoconnectome (number of synapses).

To derive the mesoconnectome, we start from the total number of synapses per layer and subsequently assign pre- and postsynatpic neurons. For the local connections, we use the connection probabilities derived by Potjans and Diesmann (2014) (Fig. 2A and Sec. “Local connectivity”) as a blueprint. The relative connection probabilities across source and target populations are kept constant, and they are only scaled by a constant factor to achieve the desired total number of local synapses in each area. The cortico-cortical connectivity on the area level is specified by DTI data from the Human Connectome Project (Goulas et al. 2016, which is based on the data from Van Essen et al. 2013; Fig. 2B and Sec. “Long-range projections”). Synapses associated with long-range projections are assigned to postsynaptic neurons according to morphological reconstructions of human neurons (Mohan et al. 2015; Fig. 2C and Sec. “Long-range projections”).

The laminar origin of long-range projections is based on predictive connectomics. Retrograde tracing data in macaque show that the laminar origin is systematically related to the cytoarchitecture (Hilgetag et al. 2019; Fig. 2D). Assuming that the same relation also holds in human, we use the fit in combination with the human cytoarchitecture to determine the laminar origin (Fig. 2E). For the laminar target, we assume the same relation between laminar origin and target as done for macaque by Schmidt et al. (2018a), for lack of layer-specific human data.

Combining these data, we arrive at a human mesoconnectome which specifies the number of synapses between excitatory and inhibitory neurons for all areas in the Desikan–Killiany parcellation on a layer- and population-specific level (Fig. 2F).

#### Connectivity validation

To validate the derived mesoconnectome, we compare it with anatomical features that were observed in other species but that were not explicitly built in.

**Fig. 3:**
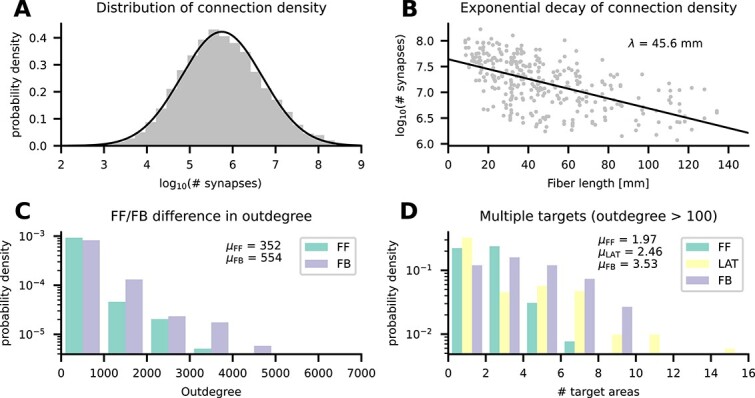
Connectivity validation. (**A**) Histogram of the number of synapses between pairs of populations (gray bars) and a log-normal fit (black line). (**B**) Logarithmic number of synapses between a pair of areas versus distance between these areas (gray symbols) and an exponential fit with decay constant $\lambda $ (black line). (**C**) Average outdegree of a neuron in any given population to any postsynaptic area in either feedforward (FF) or feedback (FB) direction. (**D**) Average number of target areas of a neuron in any given population to any postsynaptic area with average outdegree larger than $100$ in either feedforward (FF), lateral (LAT), or feedback (FB) direction.

The density of connections between areas is highly heterogeneous, spanning 5 orders of magnitude, and approximately log-normally distributed in mouse (Gămănuţ et al. 2018), marmoset (Theodoni et al. 2021), and macaque (Ercsey-Ravasz et al. 2013). Similarly, in our model the numbers of synapses between pairs of populations span 5 orders of magnitude (Fig. 3A) and they are approximately log-normally distributed. Furthermore, the connection density decays exponentially with distance in mouse (Horvát et al. 2016), marmoset (Theodoni et al. 2021), and macaque (Ercsey-Ravasz et al. 2013). In our model, the number of synapses between pairs of areas also decays exponentially (Fig. 3B) with a decay constant of $45.6\,\mathrm{mm}$. Thus, 2 salient features of tracing data are captured by our model.

Anterograde tracing data indicate that feedback axons arborize more strongly than their feedforward counterparts (Rockland 2019). This suggests a larger outdegree of feedback projections compared with feedforward projections. In our model, the average outdegree from neurons in a given population to a given target area varies systematically between feedforward and feedback projections (Fig. 3C); here, feedforward and feedback were classified based on the predicted $\mathrm{SLN}$ value (Schmidt et al. 2018a): $\mathrm{SLN}>65\%$ (feedforward), $35\%\le \mathrm{SLN}\le 65\%$ (lateral), and $\mathrm{SLN}<35\%$ (feedback). The average outdegree for feedforward inter-area connections in our model is $352$ compared with $554$ in the feedback direction. While the model preserves the biological neuron and synapse density as well as the average indegree, modeling all projections as coming from the $1\:\mathrm{mm}^{2}$ microcircuits alters the average outdegree for inter-area projections. Specifically, this multiplies the average outdegree by the ratio of source area surface to target area surface; taking this factor into account leads to an estimated biological average outdegree of $793$ in the feedforward and $1221$ in the feedback direction.

Finally, fully reconstructed axons (Winnubst et al. 2019) suggest that many projecting neurons target multiple areas. To check for such divergence in the model, we restrict ourselves to connections with an average outdegree larger than $100$. Again using the predicted $\mathrm{SLN}$ value to separate feedforward, lateral, and feedback connections, we obtain a broad distribution of the number of target areas (Fig. 3D). In addition to the larger outdegree in the feedback direction, feedback projections also target more areas: on average $3.53$ compared with $2.46$ for lateral and $1.97$ for feedforward projections.

### Micro- and macroscopic dynamics

#### Spiking activity in the base version

**Fig. 4:**
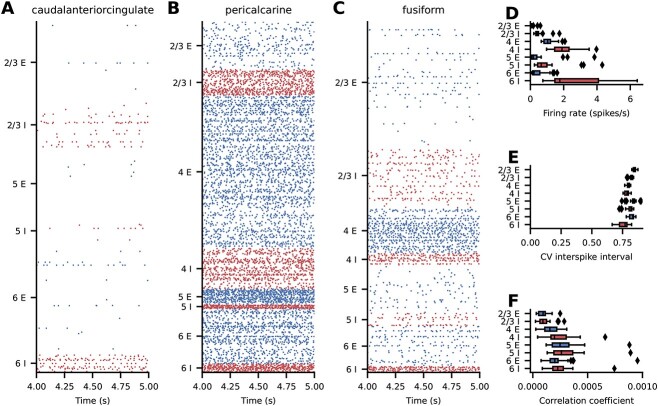
Spiking activity in the base version of the model. (**A–C**) Raster plots for 3 representative areas; subsampled to $2.5\%$ of the excitatory (blue) and inhibitory (red) neurons. (**D–F**) Layer- and population-resolved distribution of population-averaged statistics across areas; boxes show quartiles, whiskers are within $1.5$ times the interquartile range, symbols show outliers outside of the whiskers. (**D**) Firing rate. (**E**) CV ISI of neurons with at least $10$ spikes. (**F**) Pairwise correlation coefficient of a random subsample of $2000$ neurons for each population.

We first consider simulations with equal strengths of local and inter-areal synapses. The simulated spiking activity of this base version of the model is asynchronous and irregular with low firing rates across all areas (Fig. 4). There is a pronounced structure of the activity across populations, layers, and areas (Fig. 4A–C). To quantify the spiking activity further, we consider population-averaged statistics (Fig. 4D–F). The firing rate of the inhibitory neurons is higher than the firing rate of the excitatory neurons, with the highest activity in layer 6 (Fig. 4D). The activity of some excitatory populations is very low, in particular in layers 2/3 and 5 (Fig. 4D). In terms of the irregularity of the spike trains, quantified by the coefficient of variation $\mathrm{CV}$ of the interspike intervals, all populations are in the regime of $\mathrm{CV\:ISI}\approx 0.8$ (Fig. 4E), i.e. slightly more regular than a Poisson process. Lastly, the average pairwise correlation between the neurons is close to zero across all populations (Fig. 4F).

#### Comparison with experimental activity data

To obtain stronger inter-areal interactions, we increase the inter-areal synaptic weights onto excitatory neurons by the inter-areal scaling factor $\chi $ and onto inhibitory neurons by a factor $\chi _{I}\chi $, where $\chi _{I}=2$. We compare the resulting activity of the model with experimental activity data on 2 levels: on the neuron level, we use the electrophysiological recordings by Minxha et al. (2020) from human medial frontal cortex (cf. Sec. “Experimental spiking data”); on the cortex level, we use resting-state fMRI data from 19 subjects (cf. Sec. “FMRI data”). The electrophysiological data were recorded in dorsal anterior cingulate cortex and pre-supplementary motor area; we compare the data with the model activity in area caudalanteriorcingulate. The pre-supplementary motor area overlaps with our model area superiorfrontal but forms only a small part of it, so that the 2 cannot be meaningfully compared. Since the recordings are not layer- or population-specific, we combine the spike trains of all layers and populations in caudalanteriorcingulate for this analysis. In both the experimental and simulated data, we consider only neurons with at least $0.5$ spikes/s for the firing rate, and, for the irregularity, expressed as the coefficient of variation of the interspike intervals (CV ISI) and revised local variation (LvR), we consider only neurons with at least $10$ spikes in the respective interval. LvR is a measure of spike train irregularity that corrects for firing rate variations and refractoriness (Shinomoto et al. 2009). As the spike trains comprise only s of activity, we divide the 10 s of simulated activity into 5 snippets of equal length. In order to compare the experimental and simulated distributions, we calculate the Kolmogorov–Smirnov distances between them and report $1-KS_{\mathrm{dist}}$ as a measure of similarity, where $0$ means no and $1$ means perfect similarity. To obtain a proxy for the BOLD signal from our model, we use the absolute value of the area-level synaptic currents (Schmidt et al. 2018b). We compute the functional connectivity using the Pearson correlation coefficient of this BOLD proxy (simulation) or the BOLD signal (experiment). As a measure of the similarity between the modeled and empirical functional connectivity we use the Pearson correlation coefficient and the root-mean-square error (RMSE), in both cases excluding the diagonal where all values are identically one. We convert the RMSE to a similarity measure using $\exp (-\mathrm{RMSE}_{\mathrm{sim}}/\sigma _{\mathrm{exp}})$, where $\sigma _{\mathrm{exp}}$ denotes the standard deviation of the functional connectivity. We use both methods because the Pearson correlation is based on relative values and quantifies the linear relationship between the variables, while the RMSE-based measure takes into account the absolute FC strengths.

**Fig. 5:**
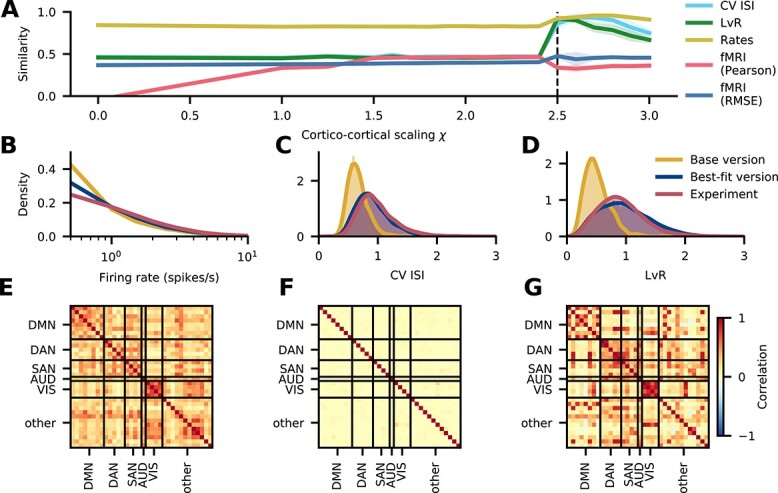
Comparison with experimental activity data. (**A**) Similarity of simulated spiking activity in area caudalanteriorcingulate to experimental spiking data (Minxha et al. 2020) recorded in medial frontal cortex and to resting-state fMRI functional connectivity (cf. Sec. “FMRI data”) as a function of the scaling parameter $\chi $ for inter-areal synaptic strengths. The vertical dashed line at $2.5$ corresponds to the chosen best-fitting version. The shaded areas represent the standard deviation over $10$ simulation runs, each with a different random seed. (**B–D**) Distribution of spiking statistics across neurons in experimental spiking data (Minxha et al. 2020) and in the simulated base and best-fitting versions: distribution of firing rates (**B**), CV ISI (**C**), and revised local variation (LvR; Shinomoto et al. 2009) (**D**). (**E–G**) Functional connectivity in the default mode network, DAN, SAN, auditory network (AUD), visual network (VIS), and the remaining areas (other). Experimental functional connectivity of the right hemisphere from fMRI recordings, averaged across 19 subjects (**E**). Simulated functional connectivity based on synaptic input currents in the base (**F**) and the best-fitting version (**G**).

Figure 5A shows how the different similarity measures depend on the inter-areal scaling factor $\chi $. The agreements of the $\mathrm{CV}\;\mathrm{ISI}$, the LvR, and the rates initially stay constant and these measures abruptly show a higher agreement at $\chi =2.5$. At $\chi $-values close to $2.5$, the network sometimes starts in a state of high activity and then, after an initial transient, settles in a lower activity state or, depending on the random seed, the network operates in a higher- or lower activity state for the same value of $\chi $ for the full simulation duration. To exclude transients due to a transition from a high- to a low-activity state, we disregard the first $2500\,\mathrm{ms}$. The distributions of the spiking activity measures continue to match the experimental data well until $\chi =2.8$. Afterwards, the similarities of the irregularity measures $\mathrm{CV}\;\mathrm{ISI}$ and LvR deteriorate. The similarity of the fMRI functional connectivity calculated using the Pearson correlation of the experimental and simulated functional connectivity matrices grows from $0.37$ to $0.47$ and then suddenly drops to $0.33$ at $\chi =2.5$, a value around which it remains. The correlation to be maximally accounted for by the model is given by the mean correlation of the experimental functional connectivities across subject pairs, which is $0.63$; this ceiling is thus not reached. On the other hand, using the RMSE, the similarity stays initially around $0.38$ and grows to $0.46$ at $\chi =2.5$, which is consistent with the behavior of the spiking activity measures. The mean RMSE-based similarity between experimental functional connectivities of different subjects is $0.59$. Thus, also in terms of this measure, the model does not fully account for the empirical FC structure in human subjects, but it comes closer than the Pearson correlation. As $\chi =2.5$ is the first point at which most measures show good agreement, we use this setting for further analysis. In the following text, we refer to this setting as “the best-fitting version.”

**Fig. 6:**
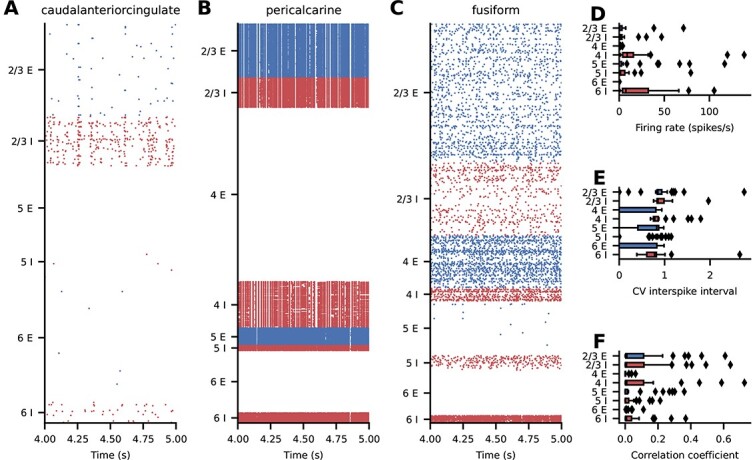
Best-fitting spiking activity of the model. (**A–C**) Raster plots for 3 representative areas; subsampled to $2.5\%$ of the excitatory (blue) and inhibitory (red) neurons. (**D–F**) Layer- and population-resolved distribution of population-averaged statistics across areas; boxes show quartiles, whiskers are within $1.5$ times the interquartile range, symbols show outliers outside of the whiskers. (**D**) Firing rate. (**E**) $\mathrm{CV}\;\mathrm{ISI}$ of neurons with at least $10$ spikes. (**F**) Pairwise correlation coefficient of a random subsample of $2000$ neurons for each population.

A closer look at the underlying statistics (Fig. 5B–D) confirms that the best-fitting version matches the experimental data better than the base version does. The firing rate distribution (Fig. 5B) is reproduced well by both the base and the best-fitting version, but the latter follows the experimental distribution slightly better. This matches the observation in Fig. 5A, where the firing rate similarity is high throughout and peaks at the best-fitting version. The $\mathrm{CV}\;\mathrm{ISI}$ (Fig. 5C) shows clear differences between the base and the best-fitting versions: in the former, the $\mathrm{CV}\;\mathrm{ISI}$ is narrowly distributed around a sub-Poissonian average; in the best-fitting version and the recordings, the $\mathrm{CV}\;\mathrm{ISI}$ is broadly distributed around a Poissonian average. These 2 distributions match almost exactly. Similar observations hold true for the LvR, where the main difference compared with the $\mathrm{CV}\;\mathrm{ISI}$ is that all distributions are slightly broader.

To facilitate the comparison of the functional connectivities, we group the areas into clusters of different resting-state networks following Kabbara et al. (2017). The experimental (Fig. 5E) and best-fitting (Fig. 5G) functional connectivities show a clear structure with increased correlations within the clusters in the resting-state networks, while the functional connectivity of the base version shows only very weak correlations (Fig. 5F). Also the enhanced correlations between the dorsal attention network (DAN) and the salience network (SAN) are well captured by the model in the best-fitting version. These improvements are captured by the RMSE-based measure, which takes into account the absolute FC values, as opposed to the Pearson correlation, which only considers the linear relationship between the empirical and simulated FC.

#### Analysis of best-fitting version

The simulated spiking activity in the best-fit version varies across areas both quantitatively and qualitatively. Generally, firing rates are higher in the best-fit version than in the base version (Fig. 6). Some areas, such as caudalanteriorcingulate (Fig. 6A) and fusiform (Fig. 6C), show low-rate uncorrelated spiking activity with brief population bursts, while some areas, such as pericalcarine, are in a state of high firing in most populations. For completeness, the raster plots of all areas are shown in the Appendix (Figs. S11, S12, S13). We consider population-averaged statistics to quantify the spiking activity on the level of the full network (Fig. 6D–F). Inhibitory neurons have higher firing rates than excitatory neurons, with the highest activities in layers IV and VI (Fig. 6D). The activity of some excitatory populations is very low, particularly in layers 2/3, 4, and 6. The irregularity of the spike trains, quantified by the $\mathrm{CV}\;\mathrm{ISI}$, is on average closer to that of a Poisson process compared with the base version, but also varies more strongly across areas (Fig. 6E). The average pairwise correlations are generally low, but reach higher values in a number of areas (Fig. 6F).

### Temporal hierarchy

**Fig. 7:**
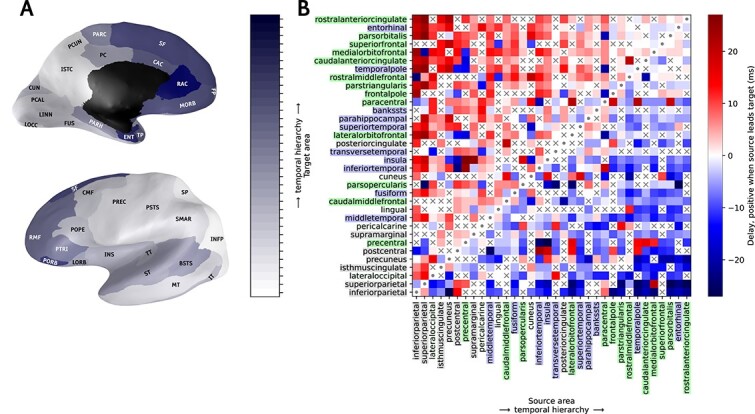
Temporal hierarchy of activity flow across the network. Inflated medial and lateral views of the brain (**A**) depict the temporal order of activation, with colors ranging from white for the most leading area to dark blue for the last. The black zone in the medial view is not part of the model. The temporal hierarchy matrix in (**B**) is based on the estimation of delays obtained as peaks in the cross-correlation function between source and target area, with the ordering of areas determined as described in the section “*Temporal Hierarchy from Model Spiking Data*”. The matrix is symmetric across the diagonal with inverse sign. Cells marked with “x” indicate delays classified as ‘undecided’. On the x- and y-axis the brain area labels are colored according to a coarse anatomical division of parietal (gray), occipital (light gray), temporal (blue) and frontal (green) lobes. Abbreviations from Table 1. Brain meshes from Winkler (2013); Bakker et al. (2015).

An important aspect of global network dynamics is the temporal relation between signals in different brain regions. An estimate of the direction of the activity flow, which we term “temporal hierarchy” (see section “*Temporal Hierarchy from Model Spiking Data’*’) in the best-fitting version of our model is shown in Fig. 7B. Its construction is inspired by Mitra et al. (2014), though they worked with fMRI data, which reveals dynamics on the scale of seconds rather than milliseconds. We see an activation pattern following the order of parietal, occipital, temporal, and frontal areas. We compare our result with the work of Dentico et al. (2014), which is based on EEG data. They look at the flow of activity under 2 conditions: visual perception and visual imagery. Their findings show that the flow of activity, in particular from the occipital lobe to the parietal gyrus, reverses when visual input is absent. This is consistent with the temporal hierarchy in our model, which has no visual input, and has the parietal cortex leading the occipital areas. In the macaque visual cortex model of Schmidt et al. (2018b), their Figs. 7D and 7G show the same pattern of parietal leading occipital regions. Furthermore, in both our model and that described in Schmidt et al. (2018b), the parietal areas are the first to become activated, identifying these as drivers of cortical spontaneous activity.

### Propagation of a single-spike perturbation

In vivo, single-neuron perturbations can affect behavior (Brecht et al. 2004; Houweling and Brecht 2008). But how does a single-neuron perturbation spread across the cortical network consisting of millions of neurons or more? We investigate this in our model comprising $3.5$ million neurons. To this end, we perturb the membrane potential of a single excitatory neuron in layer 4 in primary visual cortex (area pericalcarine) such that it exceeds the threshold and emits a spike. On the network level, this is an extremely weak perturbation. However, since spiking networks are highly sensitive to perturbations (London et al. 2010; Monteforte and Wolf 2010), even a single spike can alter the spiking pattern of the network (Izhikevich and Edelman 2008).

**Fig. 8:**
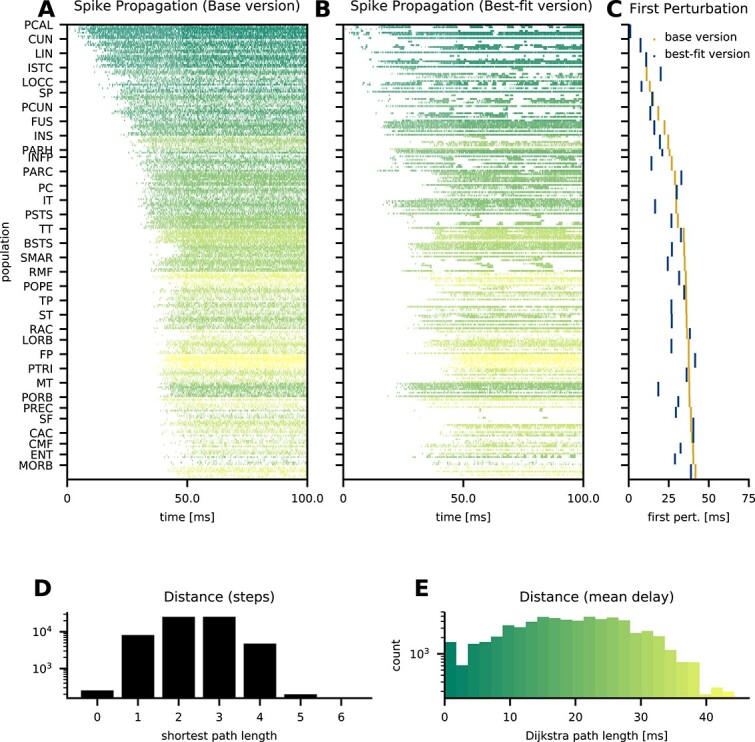
Propagation of the effects of a single spike. Binary absolute difference of spike counts per population in $0.1\,\mathrm{ms}$ bins between a perturbed and an unperturbed simulation with identical parameters and random seeds in the base version (**A**) and the best-fit version (**B**); the color quantifies the Dijkstra path length between the perturbed and the target population. Populations are ordered corresponding to the previous figures; for the scale see panel E. Timing of the first spike count difference per area (**C**) in the base version (orange) and the best-fit version (blue). Histogram of shortest path lengths between all pairs of populations in the network (**D**). Histogram of shortest path lengths weighted by the average delay between all pairs of populations in the network (**E**).

We perform 2 simulations with identical parameters and random seeds but once without and once with the single-neuron perturbation. The drawn random numbers and their total number are the same in both simulations. To quantify alterations of the spiking pattern, we count the total number of spikes of a population in $0.1\,\mathrm{ms}$ bins and compute the difference between the unperturbed and the perturbed simulation. As soon as the difference is nonzero due to an additional or missing spike, our observable is set to one. Thus, the observable quantifies the presence or absence of a spike in a given population due to the perturbation. In both the base version (Fig. 8A) and the best-fitting version (Fig. 8B), the perturbation propagates to all areas in less than $50\,\mathrm{ms}$. In the best-fitting version, the perturbation propagates even slightly faster to most areas (Fig. 8C). Presumably, the increased activity level in the best-fitting version contributes to this difference in propagation speed (Fig. 6). In the base version, the propagation time is $29.4\pm 10.9\,\mathrm{ms}$ (mean $\pm $ standard deviation); in the best-fitting version, it is $25.1\pm 10.4\,\mathrm{ms}$.

How is this fast propagation possible? Just like weighted area-level cortical graphs of mice and macaques (Bassett and Bullmore 2017), the population-level graph in our model exhibits small-world network properties (Watts and Strogatz 1998). Namely, only a small number of steps is needed to reach any node: the shortest path length between any pair of populations is between 1 and 4 and at most 5 (Fig. 8D). But the shortest path length in terms of the number of populations traversed does not account for the transmission delay, which is particularly relevant between areas. Taking also the delay into account by weighting each step with the mean delay and computing the Dijkstra path length (Dijkstra 1959), i.e. the shortest path based on the sum of the mean delays, we see that the small-world property of the network enables a Dijkstra path length below $50\,\mathrm{ms}$ for any pair of populations and below $40\,\mathrm{ms}$ for the majority of pairs (Fig. 8E). Thus, the network structure supports fast propagation at the population level. The propagation of the perturbation indeed takes place on a timescale similar to the Dijkstra path length between the perturbed population and the target population. The distribution of delays (present in both model versions) in principle allows propagation to take place even faster than this path length.

## Discussion

We aggregated data across multiple modalities, including electron microscopy, electrophysiology, morphological neuron reconstructions, and DTI, to construct a multi-scale spiking network model of human cortex. In this computational model featuring $3.5$ million neurons connected via $43$ billion synapses, each area in a full hemisphere of human cortex is represented by a millimeter-scale layer-resolved microcircuit with the full density of neurons and synapses. The model was simulated on a supercomputer, using advances in the simulation technology of NEST. We filled gaps in the data using statistical regularities found in other species, in particular to determine the laminar origins and targets of inter-areal connections. Comparisons with electrophysiological recordings from human medial frontal cortex and human fMRI reveal that the model captures aspects of both microscopic and macroscopic resting-state activity when the strength of the inter-areal synapses is increased.

### Base vs. best-fitting version

Simulations of the model with equal local and inter-areal synaptic strengths (which we refer to as the “base version” of the model) reveal a state with asynchronous and irregular activity. The activity is heterogeneous across areas, layers, and excitatory and inhibitory populations. The activity deviates from the experimental recordings in terms of both spiking activity and inter-area functional connectivity. On the single-neuron level, the distribution of the spiking irregularity in the model is more narrow than the observed one and centered in the sub-Poissonian regime. On the network level, the activity is hardly correlated between areas, which stands in stark contrast to the salient structure in the fMRI data.

To alleviate these discrepancies, we increased the synaptic weights of inter-areal connections. The increased anatomical connection strength leads to an increase in inter-areal correlations, with a modular structure similar to the experimental data. On the level of the single-neuron statistics, the increased inter-areal synaptic weights hardly affect the distribution of firing rates and irregularity until the synaptic weights reach a critical value at which the fit to the experimental data suddenly improves. This best-fitting version features not only stronger correlations between the activity in different areas but also within areas and populations. Furthermore, the firing rates, in particular in the inhibitory populations, are increased. Although the low overall firing rates and the higher inhibitory compared with excitatory rates are realistic features (Dehghani et al. 2016; Dąbrowska et al. 2021), some layers and populations of the model exhibit either seemingly excessive or nearly vanishing rates. Since recordings of spiking activity from human cortex are few and far between, a “ground truth” to compare these spike rates with is not available. Furthermore, experimental recordings may miss many neurons that do not spike within the recording window (Shoham et al. 2002; Urai et al. 2022). However, assuming that human cortical activity is like that from other species, completely silent neural populations and spike rates exceeding a few tens of spikes per second are anomalous. Besides the large variation in spike rates, a number of areas display highly synchronous activity (cf. Fig. S11–S13). To some extent, this may be less unrealistic than it appears at first sight, because vertical stripes in raster plots are emphasized when the spikes of more neurons are plotted: vertical stripes in raster plots of experimental spiking activity are less prominent than for a simulation with the same degree of synchrony where the spikes of many more neurons are shown. We previously presented such an example of simulated macaque V1 spiking activity, appearing highly synchronized upon visual inspection but matching both single-neuron spiking statistics and population activity power from experiments (Schmidt et al. 2018b). Nevertheless, the synchrony and large variations in spike rates across areas and layers in the present model are probably not yet an accurate reflection of cortical spiking activity, and remain to be addressed. One promising avenue is to enhance local balance via joint clustering of excitatory and inhibitory neurons (Pronold et al. 2024; Rostami et al. 2024). This refinement was shown to normalize spike rate distributions in a recent multi-area model of macaque cortex (Pronold et al. 2024), but is beyond the scope of this study.

### Propagation of macroscopic fluctuations and single-spike perturbations

Computational models allow one to examine questions that are hard to investigate experimentally. Here, we study how both macroscopic activity fluctuations and single-spike perturbations propagate through the network. First, we construct a “temporal hierarchy” of inter-area propagation from the ongoing activity based on the cross-correlation functions of the area-level spiking activity. The results reveal a dominant order of parietal, occipital, temporal, and then frontal areas. Parietal areas leading the activity matches findings from a model of all vision-related areas in macaque cortex (Schmidt et al. 2018b), and the predominant activation of parietal before temporal regions matches EEG findings during visual imagery (Dentico et al. 2014). Different from Schmidt et al. (2018b), occipital areas precede temporal areas, and predominantly positive rather than negative correlations are found between the frontal areas and the remainder of the network. Possible reasons for these differences include the fact that the former study only included 2 frontal areas, whereas we here model a full hemisphere; and the adjustment of the method for constructing the temporal hierarchy, where we discard oscillatory activity. Coito et al. (2019) analyzed the directed functional connectivity of spontaneous EEG and found the strongest outflows from cingulate and medial temporal regions. This appears different from our results, although their methods differ strongly from ours and they did not assign an overall propagation order across all areas. As such, our model prediction merits further investigation. In future, propagation upon stimulation of for instance primary visual cortex may also be studied, akin to Joglekar et al. (2018) and Pronold et al. (2024). These studies report, respectively, that balanced amplification and joint clustering of excitatory and inhibitory cells may aid macroscopic activity propagation through the cortical network.

Second, we use our model to track the effect of a single additional spike through the large-scale network. We find that the single-spike perturbation spreads across the entire network within less than $50\,\mathrm{ms}$, close to the lower limit imposed by the mean transmission delay between the areas along the shortest possible path. In the best-fitting version, the propagation is even faster than in the base version. The observed latencies are on the same order as visual response latencies across macaque cortex (Lamme and Roelfsema 2000), but note that single-spike perturbations may not be visible on the population level. Rapid propagation of spiking activity, whether on the single-neuron or the population level, is likely to support fast sensory processing and behavioral responses. Due to the stochastic input to the network and its sensitivity to small perturbations, the triggered spike sequences are not fixed but will differ between trials. However, signal separation and classification performance may benefit from the divergence of trajectories due to chaos (Keup et al. 2021). The stochasticity of the external drive in our model reflects the lack of knowledge about the activity of the non-modeled parts of the brain. In reality, these inputs will be more deterministic and less variable across trials, and may therefore support more reliable spike sequences. Future work may furthermore investigate whether subnetworks with strong synapses, such as those modeled for turtle cortex by Riquelme et al. (2023), can support repeatable and precisely timed spike sequence in the human cortical network.

### Delineation from other species

The approach we followed closely resembles that taken for the multi-area model of macaque vision-related cortex of Schmidt et al. (2018a, b). A notable difference compared with that model is that our best-fitting version is stable over the full length of the investigated simulations, in contrast to the metastable activity obtained there, which sometimes switched to a high-activity state after long simulation durations. In the best-fitting version, our model still exhibits a type of metastability: in some simulations, the activity is initially high and later switches to the lower activity state that matches the experimental data better and that we analyze. The increased stability of the best-fitting state in the present model compared with the macaque model and the lack of excessive network-averaged firing rates throughout the simulations provide a better match to actual brain activity.

Just like the model of Schmidt et al. (2018a, b), the present model predicts that stronger inter-areal compared with local synapses are needed to account for appreciable functional connectivity between areas, a feature that may be investigated experimentally. In our model, the inter-areal synapses are, moreover, stronger onto inhibitory than onto excitatory neurons. A similar feature was reported in mice, where interareal excitatory synaptic input to layer 2/3, but not to layer 5, parvalbumin-expressing interneurons is stronger than to pyramidal neurons (Yang et al. 2013; D’Souza et al. 2016; D’Souza and Burkhalter 2017). However, using estimates of the relative densities of excitatory and inhibitory neurons taken from cat area 17 (Gabbott and Somogyi 1986; Binzegger et al. 2004; Potjans and Diesmann 2014), we were also able to obtain good correspondence with experimental resting-state activity in simulations with very strong inter-areal synapses, equal in strength onto excitatory and inhibitory neurons (Fig. S6). In this case, stability was afforded by stronger local synapses onto inhibitory compared with excitatory cells, consistent with slice data from human cortex (Campagnola et al. 2022). In all cases, we did not need to adjust the connection densities to obtain plausible activity as done in the macaque model (Schuecker et al. 2017). This is an improvement because now the connection densities can be directly estimated from the empirical data.

A question that naturally emerges is what sets human cortex apart from that of other species in terms of the properties that determine its resting-state activity statistics. One property that differs with respect to other species is the fraction of excitatory vs. inhibitory neurons, which appears to be lower especially in human cortical layer 2/3 (Gabbott and Somogyi 1986; Sahara et al. 2012; Shapson-Coe et al. 2021; Alreja et al. 2022). Our model predicts that this reduced excitation in the supragranular layers necessitates greater inter-area coupling for the resting-state activity statistics to match the experimental data, and further leads to a slighty different pattern of functional connectivity between areas (cf. Fig. 5, Fig. S6). Future work may consider a selective increase in the occurrence of bipolar-type interneurons, which preferentially target other inhibitory neurons (Loomba et al. 2022). Further, human cortical neurons tend to be larger and have a lower count density than in other species, receiving more synapses per neuron on average (Sherwood et al. 2020; Loomba et al. 2022). This is likely to be advantageous for information processing, due to a combinatorial explosion of potential synaptic co-activations, but even the implications for resting-state activity remain to be understood. As we have also mentioned and incorporated into our model, the inter-area connectivity of human cortex is sparser because the increased surface between the gray and white matter does not make up for the increased brain volume, so that relatively fewer myelinated axons can connect the areas than in species with smaller brains (Herculano-Houzel 2009). Another prominent feature of human cortex is its large number of areas, although the increase in this number with respect to other species appears only moderate compared with the expansion of the surface area (Changeux et al. 2021). This study uses a coarse parcellation both for computational efficiency and to limit the number of unknown parameters, but future work may refine the model toward the potentially $180$ or more areas per human cortical hemisphere (Glasser et al. 2016; Amunts et al. 2020). A further aspect, not yet considered here, is the large transcriptional diversity of human cortical neurons, which putatively form hundreds of cell types (Hodge et al. 2019; Miller et al. 2019). Taking into account this extensive diversity would necessitate estimating a huge number of connection probabilities, scaling with the square of the number of cell types, which the available experimental data do not yet allow. This complexity may be gradually approached in future. Also certain electrophysiological properties differ between the cortical neurons of humans and those of other species; for instance, human layer 2/3 pyramidal cells have a smaller specific capacitance, which may to some extent be compensated by the larger size of human neurons (Eyal et al. 2018). Here, we have included distinct human-specific electrophysiological parameters for excitatory and inhibitory cells, but the investigation of further cell-type diversity and the comparison with single-neuron parameters from different species are left to future work.

### Outlook

Various assumptions and approximations flow into the model definition. For instance, with the modeled inhibitory postsynaptic potentials being 5 times as large as excitatory ones, the relative strength of inhibitory synapses is rather high in the model, in vitro recordings suggesting a factor closer to $1$ (Campagnola et al. 2022). However, reducing the IPSP-to-EPSP ratio even to a value of $2$ does not allow adequate reproduction of the observed microscopic and macroscopic activity statistics (see Fig. S3). A possible resolution to this apparent inconsistency is that cortical circuits achieve effective inhibition via other factors than simply PSP size, such as more precisely attuned inhibition at the level of small subcircuits, individual neurons, or even dendritic branches (Xue et al. 2014; Arkhipov et al. 2018; Pronold et al. 2024; Rostami et al. 2024; Znamenskiy et al. 2024; Horton et al. 2024). For simplicity and model robustness, we defined the synaptic strengths via only a few parameters; in reality, synaptic strengths are diverse, for instance having laminar specificity, and the properties of synapses conveying feedforward and feedback signals are likely to differ (Germuska et al. 2006; Bastos et al. 2012; Self et al. 2012). In addition, the electrophysiological properties of individual neurons are known to be distributed, as characterized in detail in the Allen Cell Types Database (Teeter et al. 2018). However, using distributions based on the human neuron parameters provided by the Allen Cell Types Database leads to a worse fit to the experimental data compared with using the mean values only (see Fig. S4). A possible reason is that, in reality, intrinsic neuron parameters and input strengths are attuned to each other, preventing neurons with high intrinsic excitability from being strongly driven (Joseph and Turrigiano 2017). Another example is that we assumed the fraction of inter-areal plus subcortical connections to equal the fraction of white-matter connections; however, cortical areas, especially adjacent ones, may also be connected to some extent via the gray matter (Vandevelde et al. 1996; Anderson and Martin 2009). Furthermore, the synaptic time constants for excitatory and inhibitory connections are taken to be equal in the model, whereas these have been found to differ in nature (Spruston et al. 1995; Salin and Prince 1996; Angulo et al. 1999; Gupta et al. 2000). Using longer inhibitory than excitatory time constants, we are still able to obtain a close fit to the experimental activity data when also adjusting the membrane time constants and more strongly scaling the long-range synaptic strengths (see Fig. S5). In terms of in- and outdegrees of long-range projections, our model preserves the mean indegree but alters the mean outdegree by the ratio of source area surface to target area surface. The outdegree might affect the strength of the correlations; furthermore, the activity of the projecting neurons might be more correlated since they are assumed to be all within the same microcircuit. Further research may incorporate more realistic spatial divergence and convergence of connections along the cortical surface.

Besides qualitative approximations made in the model, detailed parameter values may also be updated in future, as additional data for human cortex are becoming available. For instance, layer- and cell-type-specific connection probabilities, synaptic strengths, and parameters of synaptic dynamics were recently measured in acute slices of human frontotemporal cortex (Campagnola et al. 2022). Furthermore, the recent electron microscopic reconstruction of a millimeter-scale fragment of human temporal cortex (Shapson-Coe et al. 2021) delivers layer- and cell-type-specific local connectivity data that may be used to adjust the microcircuit connectivity used here. The neuron morphologies used here (Mohan et al. 2015, 2023) have important selection effects, being taken from temporal cortex and neurons having to be relatively free of cutting artifacts to be selected for reconstruction, which will tend to favor neurons with relatively small apical dendritic trees. These selection effects may gradually be overcome as new data become available. Enabling further model refinement, a number of valuable resources and results have recently been published, detailing various aspects of histology, immunohistochemistry (Alkemade et al. 2022), transcriptomics (Jorstad et al. 2023; Siletti et al. 2023), and depth-resolved fMRI (Pais-Roldán et al. 2023) of the human brain. Furthermore, detailed human cytoarchitecture and receptor densities are gathered in the BigBrain (Amunts et al. 2013; Wagstyl et al. 2020; Zachlod et al. 2023), and are still being complemented with new measurements. These data follow the Julich-Brain parcellation (Amunts et al. 2020), which is more fine-grained than the Desikan–Killiany parcellation used here. Thus, the data may be leveraged either by finding an appropriate mapping between parcellations or by increasing the granularity of the model.

Experimental functional connectivity is not stationary but exhibits slow fluctuations (Deco et al. 2011). Currently, our model does not exhibit dynamics on such long timescales; we hypothesize that additional slow processes like spike-frequency adaptation, short-term plasticity, or neuromodulation are necessary to this end. Furthermore, the absence of slow activity may lead to an overestimation of the correlations in the functional connectivity estimation when applying the Balloon–Windkessel model or low-pass filtering the signal. To avoid that, we opted to base our fMRI BOLD proxy directly on the summed synaptic inputs. However, it should be noted that a direct comparison of the estimated absolute values with experimental data may not be ideal since we consider shorter timescales in our measure. Therefore, other methods should be explored in the future to account for these issues.

Our model provides a starting point for investigating cortical processes including adaptation, plasticity, and neuromodulation via simulation. It enables in silico studies of the multi-scale dynamics of the human cerebral cortex and the information processing it supports, from the level of spiking neurons to that of interacting cortical areas. To facilitate such further studies, the source code is publicly available at https://zenodo.org/doi/10.5281/zenodo.13711671.

## Supplementary Material

HumanMultiScaleModel_appendix_suppl_bhae409

HumanMultiScaleModel_latex
