# Supplementary material for: Multi-scale spiking network model of human cerebral cortex
Source: Cereb Cortex. 2024 Oct 18;34(10):bhae409. doi: 10.1093/cercor/bhae409 (PMC11491286; doi:10.1093/cercor/bhae409)
Supplement: HumanMultiScaleModel_appendix_suppl_bhae409 [file humanmultiscalemodel_appendix_suppl_bhae409.pdf]

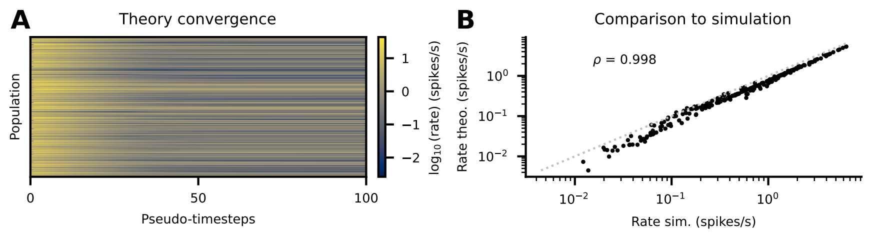

Fig. S1. Mean-field theory. (**A**) Firing rates of all populations across the pseudo-timesteps used to find a self-consistent solution. (**B**) Comparison of rates predicted by mean-field theory with empirical rates from a simulation.

Appendix

Mean-Field Theory

While developing the model, it was often beneficial to have a prediction of the activity without performing a computationally demanding full-scale simulation. To this end, we employed the mean-field theory developed by Amit, Brunel (1997) in combination with the extension to exponential postsynaptic currents derived by Fourcaud, Brunel (2002). Within this theory, the input to a neuron in population A is approximated as a Gaussian white noise with mean μ_A and noise intensity σ_A^2. The main assumptions of this theory are that the inputs are uncorrelated, that the temporal structure of the input can be neglected, that a Gaussian approximation of its statistics is valid, that the delays can be neglected, and that the variability of the neuron parameters and synaptic weights can be neglected.

Despite the simplifying assumptions, the theory provides a reliable prediction of the average firing rates if the network is in an asynchronous irregular state (Fig. S1). The remaining deviations are likely a consequence of the neglected variabilities. Thus, the theory allows for rapid prototyping without the need for high-performance computing resources. However, note that the theory does not capture the model activity well for the best-fit version of the model, as the Gaussian white noise assumption does not apply there. Thus, the simulations are the only way to determine the model activity in that case.

Sanity Checks

As sanity checks regarding the influence of the inferred connectivity, we discuss three controls: (1) Removing both long-range and intra-area connections from the model, (2) removing the intra-areal connectivity, and (3) removing all long-range projections from the model. In all three cases, we keep the external drive and remove only the model-internal synapses. The control (1) reduces to a set of isolated neurons in the mean-driven regime, see Sec. “Further Model Specifications”. Adding the inter-areal ones—which are all excitatory—for control (2) results in divergent activity due to excitatory loops. Control (3) corresponds to the case of $\chi = 0$ in Fig. 5 with the activity shown in Suppl. Fig. S8 to S10. The network activity pattern is similar to that observed in the base version, Fig. 4, but with a lower firing rate. Thus, all aspects of the connectivity strongly influence the dynamics.

Supplemental Tables

Supplemental Figures

Table S1. Summary of data sources used in the model construction and comparative analysis

Data modality	Sources
Layer-resolved neuronal volume densities and layer thicknesses	Von Economo (2009)
Mapping areas from von Economo and Koskinas parcellation to Desikan-Killiany	Goulas et al. (2016)
Layer-resolved relative size of excitatory and inhibitory populations from electron-microscopy based reconstruction of cortical tissue in the human temporal lobe	Shapson-Coe et al. (2024)
Volume density of synapses	Cano-Astorga et al. (2021)
Fraction of local connections	Herculano-Houzel et al. (2010); Herculano-Houzel (2009); Markov et al. (2011); Collins et al. (2010)
Decay constant for local spatial connection probability	Packer, Yuste (2011); Perin et al. (2011)
Relative indegrees for local microcircuit connectivity	Potjans, Diesmann (2014)
Processed DTI data to define area-level long-range connectivity	Goulas et al. (2016), which is based on Van Essen et al. (2013)
Fraction of supragranular labeled neurons	Schmidt et al. (2018), which is based on data from Markov et al. (2014a)
Anterograde tracing from non-human primates	Schmidt et al. (2018), which is based on the CoCoMac database (Stephan et al., 2001)
Probability of a long-range postsynaptic target neuron being excitatory or inhibitory	Schmidt et al. (2018), which is based on the data by Binzegger et al. (2004)
Layer-resolved soma position of targeted excitatory neurons for long-range connections	Mohan et al. (2015, 2023)
Neuron model parameters	Teeter et al. (2018); Berg et al. (2021)
Average delay for long-range inter-areal connections	Conduction velocity from Girard et al. (2001); fiber length from Goulas et al. (2016)
Spiking data from medial frontal cortex used to validate spontaneous firing activity	Minxha et al. (2020)
Magnetic resonance imaging data used to compare the functional connectivity matrices	Original data as described in Subsection 2.2.3

Table S2. Heuristics used for model construction, with consideration of possible extensions (Part 1)

Feature	Heuristic	Argument	Starting points for extensions
Neuron classes	Neurons are divided into only two classes: one excitatory and one inhibitory.	This simplification is due to the lack of fine-grained connectivity data for neuron subtypes.	Diverse human cell types are reported (Berg et al., 2021; Hodge et al., 2019; Miller et al., 2019) and could be taken into account.
Synapses	The volume density of synapses is approximated as constant across areas.	Studies in primates, including humans, show that the synapse density in the neocortex is relatively constant across areas and species (DeFelipe et al., 2002; Sherwood et al., 2020; O'Kusky, Colonnier, 1982; Cragg, 1967; Harrison et al., 2002).	Synaptic density variability found in specific cortical regions could be included (Cano-Astorga et al., 2021). In addition, gender specificity of synaptic density could be incorporated (Alonso-Nanclares et al., 2008).
Synapses	All synapses are static with a transmission probability of 100%.	For simplicity, this assumption ignores the potential effects arising from synapse dynamics.	Future extensions can include a probability of transmission failures and different types of synaptic plasticity (see, e.g., Markram et al., 2015; Jiang et al., 2023).
Population sizes	The laminar subdivisions are simplified into four layers 2/3, 4, 5, and 6. Layer 1 is ignored.	Layers 2 and 3 are combined due to their similar connectivity and comparable response properties. Layer 1 has a low neuron density compared to the other layers.	Recent studies show data related to the composition and role of human layer 1 (Boldog et al., 2018; Schuman et al., 2021; Chartrand et al., 2023), which can be used to update our model.
Population sizes	The layer thicknesses from von Economo and Koskinas parcellation are mapped to Desikan-Killiany regions by averaging corresponding areas between parcellations.	Layer thickness is not available in the Desikan-Killiany parcellation. Our mapping reflects cytoarchitectonic similarity, averaging values across similar brain regions.	Laminar thickeness from BigBrain 3D atlas (Wagstyl et al., 2020) can be used to improve the model.
Population sizes	The percentage of excitatory neurons per layer is kept constant across areas.	This is a simplification based on a lack of comprehensive area-specific data.	Refinements can incorporate area-specific data. For instance, Jorstad et al. (2023) show E:I ratios for eight human cortical regions that include primary motor, somatosensory, auditory, visual, and association areas.
Population sizes	Non-simulated neurons are replaced by a stochastic drive in the form of Poisson spike trains.	Comprehensive data on the activity of the non-modeled parts of the brain are not available. Furthermore, this simplification limits the number of parameters.	A recent study describes spontaneous cortical activity as stereotyped and non-Poissonian (Swindale et al., 2023). Different distributions could be tested in the model.
Local connectivity	We assume the spatial connection probability to be a spatially homogeneous three-dimensional exponential distribution.	This is consistent with experimental studies reporting a three-dimensional exponential fall-off in connection probability (Perin et al., 2011; Maksimov et al., 2018).	A future extension can make the spatial decay constant population-specific (Reimann et al., 2017; Senk et al., 2023) and take patchy connectivity into account.
Local connectivity	The Potjans and Diesmann (2014) model is used as a blueprint to define numbers of internal synapses per pair of populations.	This aligns with the canonical microcircuit concept (Douglas et al., 1989; Douglas, Martin, 2004) due to limited area-specific data especially for human cortex.	Reconstructions derived from human tissue fragments scanned with serial section electron microscopy (Shapson-Coe et al., 2024) promise to deliver example synapse counts between local circuit neurons.
Local connectivity	External indegrees from Potjans and Diesmann (2014) determine the relative external indegrees for the different populations.	This simplification is due to the lack of quantitative data about the local connectivity beyond the scale of a $1\,\mathrm{mm}^2$ scale column.	Since reconstructions based on electron microscopy are strongly limited in their volume due to the necessary postprocessing, similar simplifications seem necessary for the foreseeable future.

Table S3. Heuristics used for model construction, with consideration of possible extensions (Part 2)

Feature	Heuristic	Argument	Starting points for extensions
Long-range connectivity	All inter-areal connections are confined to the 1 mm^2 areas represented in our model.	Given the absence of explicit spatial dependence in our microcircuits, we adopt a simplified approach to modeling inter-areal connections.	Information on spatial divergence and convergence of inter-areal connections may be taken from studies by Colby et al. (1988); Salin et al. (1989); Gattass et al. (1997); Markov et al. (2014b).
Long-range connectivity	All inter-areal connections are excitatory.	The simplification aligns with the prevalent excitatory nature of inter-areal projections.	Studies conducted in various species, including monkeys (Tomioka, Rockland, 2007) and other animals (McDonald, Burkhalter, 1993; Gonchar et al., 1995; Fabri, Manzoni, 1996, 2004; Tomioka et al., 2005; Pinto et al., 2006; Higo et al., 2007; Urrutia-Piñones et al., 2022), have reported a relatively small proportion of GABAergic long-range connections.
Long-range connectivity	All connections between different cortical areas are treated as white-matter connections.	Long-range connections are predominantly mediated by myelinated white matter tracts.	Cortical areas, especially adjacent ones, may also be connected to some extent via the gray matter (Vandevelde et al., 1996; Anderson, Martin, 2009).
Long-range connectivity	We neglect both subcortical and inter-hemispheric projections, and treat all nonlocal synapses as belonging to intra-hemispheric inter-areal projections.	This simplification results from low presynaptic neuron percentages in subcortical structures (1%) (Markov et al., 2011) and a limited fraction of contralateral inter-areal projections (Dehay et al., 1988; Barbas et al., 2005; Rosen, Halgren, 2022).	The fraction of contralateral projections is regionally specific as reported by (Ruddy et al., 2017), and it could be incorporated to our model.
Long-range connectivity	We use regularities derived from macaque data based on retrograde tracing (Markov et al., 2014b,a; Schmidt et al., 2018) with human neuron densities to arrive at estimates of the layer-specificity of the presynaptic neurons (laminar source patterns).	This is due to the lack of relevant human-specific data.	Laminar source patterns specific to human cerebral cortex could be constructed using polarized light imaging (Axer et al., 2011) from histological brain sections or data from postmortem tracing (Galuske et al., 2000; Tardif, Clarke, 2001; Seehaus et al., 2013).
Long-range connectivity	The SLN level is used as an indicator of the type of laminar termination pattern.	This follows the observed statistical relationship between laminar sources and termination patterns as documented in combined anterograde and retrograde tracing studies gathered from the CoCoMac database (Schmidt et al., 2018).	Similar to source patterns, human-specific laminar target patterns could be obtained using polarized light imaging (Axer et al., 2011) from histological brain sections or data from postmortem tracing (Galuske et al., 2000; Tardif, Clarke, 2001; Seehaus et al., 2013).
Long-range connectivity	All inhibitory postsynaptic neurons are in the same layer as their synapses.	The dendritic tree of most inhibitory is confined to a single layer (Gouwens et al., 2019).	The morphologies of inhibitory neurons available through the Allen Cell Types Database (http://celltypes.brain-map.org/) could be used to quantify the location of the dendritic tree.
Long-range connectivity	The fraction of long-range synapses equals the fraction of long-range projecting neurons.	A preliminary analysis of reconstructed axons (Winnubst et al., 2019) corroborates the assumption.	For mice, fully reconstructed axons are available (Winnubst et al., 2019) which could be used as a starting point for a detailed analysis.

Table S4. Number of neurons per population for all areas of the model.

Area	2/3 E	2/3 I	4 E	4 I	5 E	5 I	6 E	6 I
bankssts	39130	21070	17380	4620	20280	5720	14534	2366
caudalanteriorcingulate	13179	7096	0	0	11954	3372	18297	2979
caudalmiddlefrontal	26536	14289	0	0	9262	2612	13207	2150
cuneus	35311	19014	18249	4851	11466	3234	10954	1783
entorhinal	15987	8608	0	0	4727	1333	6505	1059
frontalpole	22425	12075	18960	5040	12272	3461	18060	2940
fusiform	42250	22750	15800	4200	16575	4675	13868	2258
inferiorparietal	25350	13650	27650	7350	7722	2178	22188	3612
inferiortemporal	22912	12338	17064	4536	31200	8800	36120	5880
insula	24570	13230	19552	5197	15113	4262	17823	2902
isthmuscingulate	20950	11280	10902	2898	14820	4180	17350	2824
lateraloccipital	32110	17290	0	0	10920	3080	10084	1642
lateralorbitofrontal	15600	8400	10665	2835	16380	4620	17200	2800
lingual	36584	19699	17959	4774	14404	4063	12148	1978
medialorbitofrontal	16071	8654	4148	1102	11290	3184	12664	2062
middletemporal	22912	12338	17064	4536	31200	8800	36120	5880
paracentral	42157	22700	51251	13624	7342	2071	15741	2562
parahippocampal	29315	15785	0	0	14332	4042	0	0
parsopercularis	24635	13265	8058	2142	10733	3027	16340	2660
parsorbitalis	19500	10500	0	0	16322	4603	14216	2314
parstriangularis	20703	11148	12166	3234	6388	1802	16706	2720
pericalcarine	29250	15750	93694	24906	9477	2673	33540	5460
postcentral	22975	12371	92430	24570	8190	2310	17200	2800
posteriorcingulate	21176	11402	16649	4426	12434	3507	15960	2598
precentral	24818	13363	0	0	10608	2992	21500	3500
precuneus	28708	15458	22910	6090	8112	2288	13416	2184
rostralanteriorcingulate	13179	7096	0	0	11954	3372	18297	2979
rostralmiddlefrontal	22539	12136	12087	3213	8268	2332	13969	2274
superiorfrontal	24938	13428	7149	1900	8788	2479	15255	2483
superiorparietal	28708	15458	22910	6090	8112	2288	13416	2184
superiortemporal	30225	16275	14220	3780	15600	4400	17415	2835
supramarginal	44460	23940	32390	8610	13162	3712	19350	3150
temporalpole	22620	12180	5925	1575	21840	6160	33540	5460
transversetemporal	45695	24605	51942	13807	19500	5500	16770	2730

Table S5. Average number of incoming model-internal local synapses per neuron for all areas of the model, rounded to the nearest integer.

Area	2/3 E	2/3 I	4 E	4 I	5 E	5 I	6 E	6 I
bankssts	10214	11451	2853	6936	11376	6934	3778	3579
caudalanteriorcingulate	16103	19676	-	-	23988	15444	12523	12739
caudalmiddlefrontal	17839	20438	-	-	18289	10829	6659	7637
cuneus	8637	9345	2510	5974	8344	4843	2687	2664
entorhinal	28485	32404	-	-	27770	16209	9336	11016
frontalpole	11743	12655	5223	11209	13152	7893	5824	5419
fusiform	10859	12042	2743	7019	11130	6611	3573	3590
inferiorparietal	15112	15407	7717	15954	14446	8119	7082	6762
inferiortemporal	9183	10914	4694	10267	14961	9817	7499	6717
insula	13133	14363	5522	11916	15282	9316	6395	5841
isthmuscingulate	12481	14161	4646	10847	15665	9758	6744	6380
lateraloccipital	10705	12287	-	-	10780	6397	3265	3769
lateralorbitofrontal	10101	11734	4466	9864	14684	9457	6679	5991
lingual	9284	10175	2661	6379	9460	5599	3084	3003
medialorbitofrontal	15659	18258	4673	12208	20079	12615	8426	8280
middletemporal	9183	10914	4694	10267	14961	9817	7499	6717
paracentral	7082	7055	3303	6504	5995	3250	2209	1961
parahippocampal	8809	10408	-	-	9803	6095	-	-
parsopercularis	16861	18816	5038	13011	18170	10842	7510	7768
parsorbitalis	15615	19124	-	-	22085	14288	8690	8567
parstriangularis	16906	17994	6652	15503	16509	9412	7778	8036
pericalcarine	4294	3913	3436	5990	3947	2150	2109	1615
postcentral	5956	5310	4918	8154	5399	2949	2498	1598
posteriorcingulate	13088	14265	5542	12016	15025	9110	6415	5929
precentral	20536	23666	-	-	22793	13659	10680	11920
precuneus	14134	14776	5494	11887	13184	7470	4998	4806
rostralanteriorcingulate	16103	19676	-	-	23988	15444	12523	12739
rostralmiddlefrontal	16239	17572	5655	13348	16424	9575	6747	6810
superiorfrontal	17625	19515	4914	13109	17893	10471	7263	7770
superiorparietal	14134	14775	5494	11887	13183	7469	4998	4806
superiortemporal	12469	13894	4008	9624	13960	8459	5388	5237
supramarginal	11490	12115	4214	9289	10835	6180	3978	3851
temporalpole	13613	16240	5599	14080	20199	12963	10713	10537
transversetemporal	7846	8164	3475	6872	7908	4627	2783	2330

Table S6. Average number of incoming long-range synapses per neuron for all areas of the model, rounded to the nearest integer.

Area	2/3 E	2/3 I	4 E	4 I	5 E	5 I	6 E	6 I
bankssts	2720	663	1300	417	1666	905	4901	3084
caudalanteriorcingulate	11280	4191	-	-	1316	873	1960	1671
caudalmiddlefrontal	4648	1535	-	-	2970	1908	5758	5293
cuneus	2049	260	1642	1783	1351	381	3547	1776
entorhinal	12207	4854	-	-	246	0	45	0
frontalpole	3834	671	838	220	2071	797	4038	2315
fusiform	2715	709	1380	527	1672	918	5194	4019
inferiorparietal	4003	1022	1059	770	4281	2248	4661	4168
inferiortemporal	4087	985	1452	587	1443	783	3587	2891
insula	3917	758	1357	886	2110	960	4850	3282
isthmuscingulate	4084	866	2585	2108	1784	1012	3396	2545
lateraloccipital	2766	903	-	-	1593	968	4948	4461
lateralorbitofrontal	3728	690	1202	463	1361	584	4300	3105
lingual	2324	329	1574	1408	1489	482	4129	1934
medialorbitofrontal	4962	648	12467	16077	1386	505	2453	1881
middletemporal	4111	929	1406	493	1462	739	3646	2727
paracentral	2350	358	270	52	2363	788	3485	1923
parahippocampal	3548	708	-	-	1795	1252	-	-
parsopercularis	4488	1111	3135	2014	2380	1133	4890	4015
parsorbitalis	8842	3278	-	-	925	587	2607	2310
parstriangularis	4826	1284	1855	1155	3504	1883	3746	3156
pericalcarine	2556	348	128	0	2580	905	2064	1041
postcentral	4411	539	184	31	3477	1433	4260	2380
posteriorcingulate	4251	770	1728	1449	2016	901	3789	2433
precentral	8695	3349	-	-	1901	1201	2374	2127
precuneus	3221	664	1727	1861	2892	1273	5040	4228
rostralanteriorcingulate	8713	2954	-	-	2211	1514	3387	2898
rostralmiddlefrontal	4584	1079	1866	1043	2906	1479	4372	3175
superiorfrontal	4378	1042	4206	3286	2936	1435	4556	3509
superiorparietal	3197	688	1816	2016	2830	1318	4849	4381
superiortemporal	3258	796	2287	2031	1687	895	4102	3525
supramarginal	2962	450	1314	1243	2133	631	4266	2329
temporalpole	5529	928	12155	14462	1632	732	2657	2184
transversetemporal	2531	394	346	0	1650	555	4820	2127

Table S7. Number of incoming external synapses per neuron for all areas of the model. These numbers are multiplied by the rate of individual Poisson inputs to obtain the total Poisson input rate to each neuron.

Area	2/3 E	2/3 I	4 E	4 I	5 E	5 I	6 E	6 I
bankssts	3735	3502	4902	4435	4669	4435	6770	4902
caudalanteriorcingulate	6642	6227	-	-	8303	7888	12039	8718
caudalmiddlefrontal	6901	6470	-	-	8626	8195	12508	9058
cuneus	3001	2814	3939	3564	3752	3564	5440	3939
entorhinal	11286	10581	-	-	14107	13402	20456	14813
frontalpole	3843	3603	5044	4564	4804	4564	6966	5044
fusiform	3949	3703	5184	4690	4937	4690	7158	5184
inferiorparietal	4628	4339	6074	5496	5785	5496	8388	6074
inferiortemporal	3799	3562	4986	4511	4749	4511	6886	4986
insula	4426	4149	5809	5256	5532	5256	8022	5809
isthmuscingulate	4442	4164	5830	5275	5552	5275	8051	5830
lateraloccipital	4479	4199	-	-	5599	5319	8118	5879
lateralorbitofrontal	3869	3627	5078	4594	4836	4594	7012	5078
lingual	3277	3073	4302	3892	4097	3892	5940	4302
medialorbitofrontal	5884	5516	7722	6987	7355	6987	10664	7722
middletemporal	3799	3562	4986	4511	4749	4511	6886	4986
paracentral	2230	2090	2927	2648	2787	2648	4041	2927
parahippocampal	4626	4337	-	-	5782	5493	-	-
parsopercularis	5793	5431	7604	6879	7242	6879	10500	7604
parsorbitalis	6730	6310	-	-	8413	7992	12199	8834
parstriangularis	5297	4966	6952	6290	6621	6290	9600	6952
pericalcarine	1470	1378	1929	1745	1837	1745	2664	1929
postcentral	2159	2024	2834	2564	2699	2564	3913	2834
posteriorcingulate	4368	4095	5733	5187	5460	5187	7917	5733
precentral	7428	6964	-	-	9285	8821	13464	9750
precuneus	4512	4230	5922	5358	5640	5358	8179	5922
rostralanteriorcingulate	6642	6227	-	-	8303	7887	12039	8718
rostralmiddlefrontal	5307	4975	6965	6302	6633	6302	9618	6965
superiorfrontal	6015	5639	7895	7143	7519	7143	10903	7895
superiorparietal	4512	4230	5923	5359	5641	5359	8179	5923
superiortemporal	4335	4064	5689	5147	5418	5147	7856	5689
supramarginal	3724	3491	4887	4422	4655	4422	6749	4887
temporalpole	5391	5054	7076	6402	6739	6402	9772	7076
transversetemporal	2573	2413	3378	3056	3217	3056	4664	3378

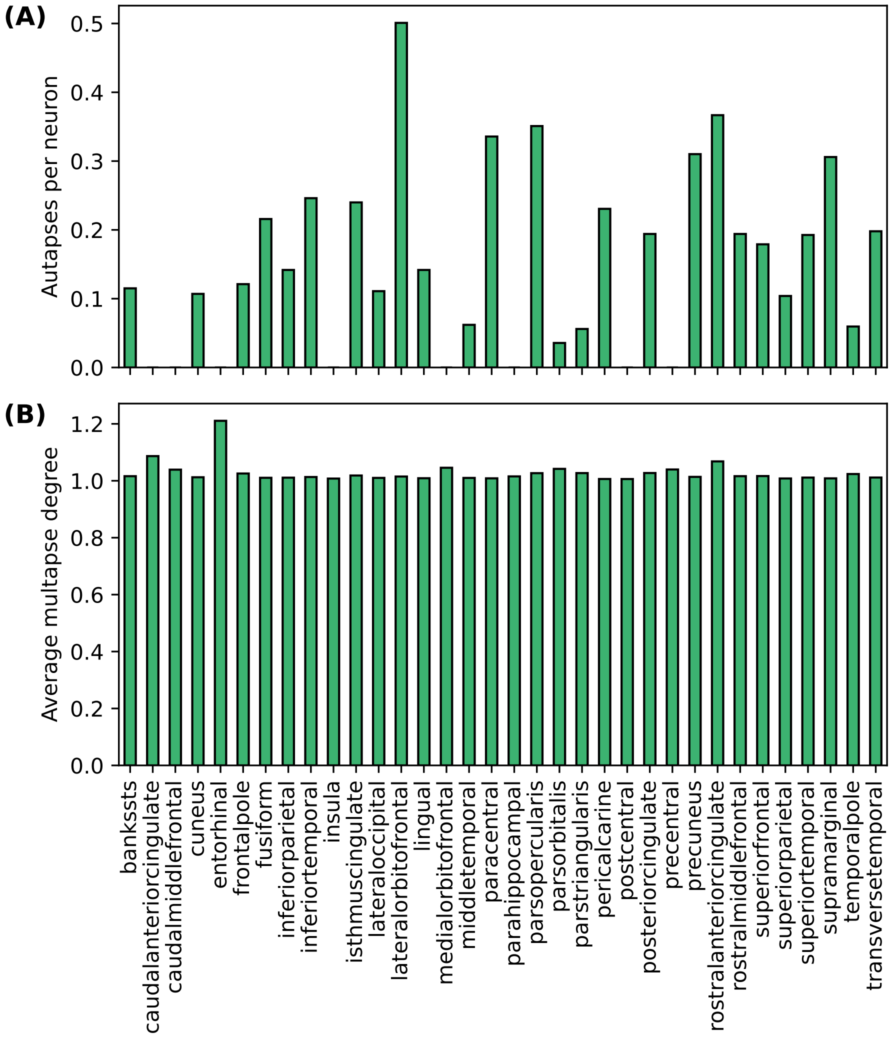

Fig. S2. Average number of autapses (self-synapses) per neuron **(A)** and average number of synapses between a pair of connected neurons (multapses) **(B).** The average multapse degrees are obtained taking into account all source populations both within and outside the given target area. They are first calculated for each population within the given target area and then averaged across those populations. The average numbers of autapses are similarly obtained by first computing them for each population within an area and then averaging across those populations.

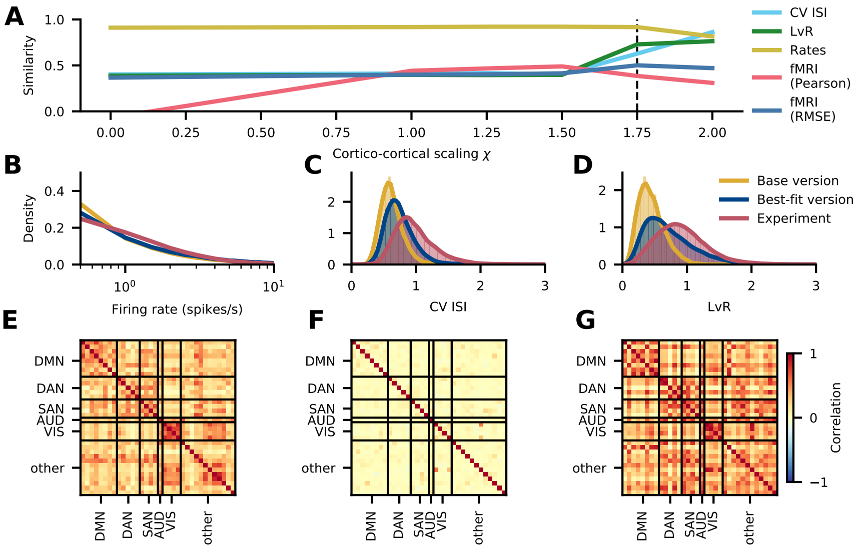

Fig. S3. Scaling plot with $g = -2$. Simulations abruptly fail at $\chi > 2$ and reach a high-activity state that cannot be simulated in a reasonable time. The vertical dashed line at $\chi = 1.75$ corresponds to the chosen best-fit version where the agreement with experimental data is maximized. The similarities of the CV ISI, LvR, and rates stay flat across most of the range of χ while the fMRI Pearson correlation shows a mostly upward trend **(A)**. The match of the firing rates **(B)**, CV ISI **(C)** and LvR **(D)** to the experimental data is better in the best-fit version compared to the base version. The functional connectivity in the base version is uniformly weak **(F)**, while it is more differentiated in the best-fit version **(G)**.

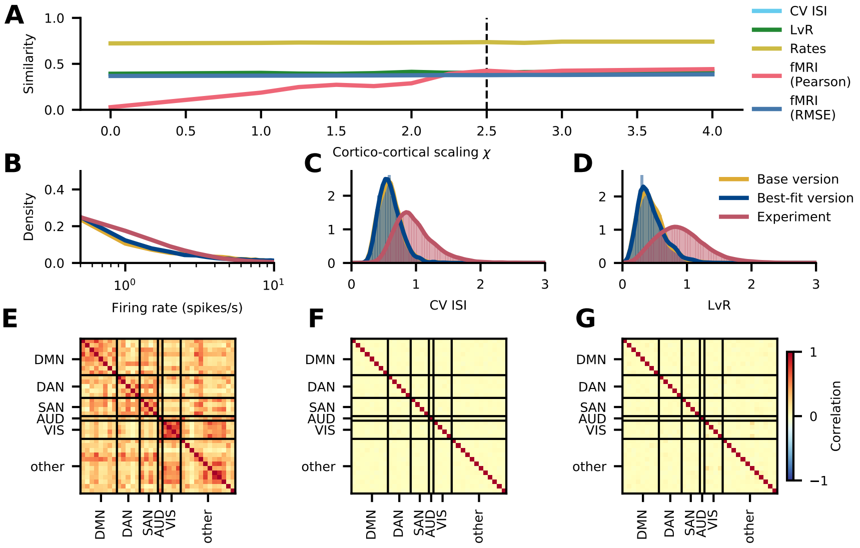

Fig. S4. Scaling plot with neuron parameters distributed according to the values for human neurons from the Allen Cell Types Database (`https://celltypes.brain-map.org/`; cf. Further Model Specifications). As this realization of the model does not show a unique best-fit solution, the vertical line at $\chi = 2.5$ shows the chosen best-fit version, similar to the original model. While the fMRI Pearson correlation benefits from increasing the inter-areal scaling factors, all other similarities stay constant. Simulations at $\chi > 4$ reach a high-activity state that cannot be simulated in a reasonable time **(A)**. The rates are explained equally well in the base version and the best-fit version, but less well than in the model without distributed neuron parameters (cf. Fig. 5) **(B)**. Neither the CV ISI **(C)** nor the LvR **(D)** is well explained in either the base or the best-fit version. The functional connectivity in both the base version **(F)** and the best-fit version **(G)** is weak, unlike the experimental functional connectivity **(E)**, although the Pearson similarity measure indicates that the best-fit version captures the structure of the functional connectivity better.

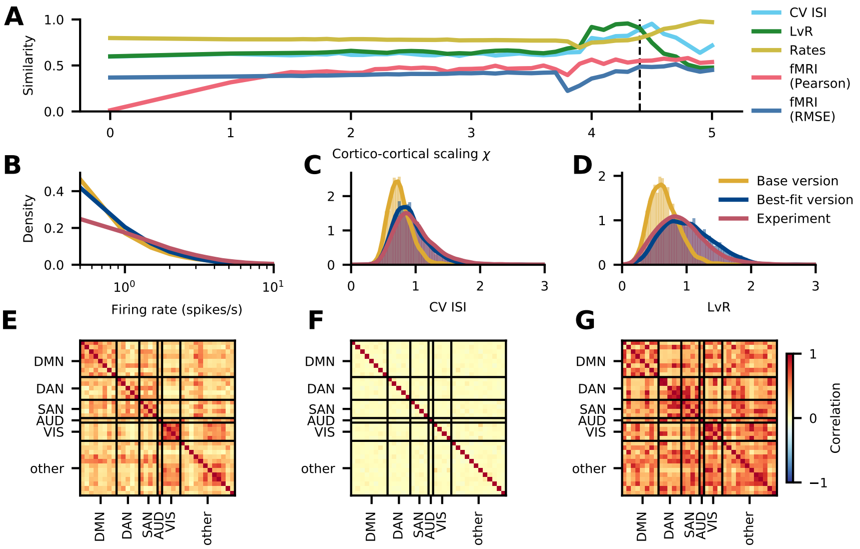

Fig. S5. Scaling plot with different synaptic time constants, $\tau_{s,E} = 2$ and $\tau_{s,I} = 4$. The membrane time constants were also adjusted to $\tau_{m,E} = 10$ and $\tau_{m,I} = 7$. The changes in the time constants directly affect the PSPs; therefore, we multiplied the PSPs onto inhibitory neurons by a factor of 10/7. With these adjustments, we were able to obtain a similar best-fit solution as in the original model, but with a stronger inter-areal scaling factor, indicated by the vertical line at $\chi = 4.4$ in **(A)**. The similarities of rates, CV ISI, LvR, and functional connectivity remain roughly constant for cortico-cortical scaling factors between 1.0 and 3.6. At the best-fit point, all similarities—except for LvR—show a slight increase. Beyond this point, the rate similarity continues to increase, while the other measures either decrease or remain constant. For firing rates above 1 spike/s, both the base and best-fit versions match the experimental data well **(B)**. The CV ISI **(C)** and LvR **(D)** in the best-fit version closely align with the experimental data. Similarly to the original model, the experimental functional connectivity **(E)** is better replicated in the best-fit version **(G)** compared to the base version **(F)**.

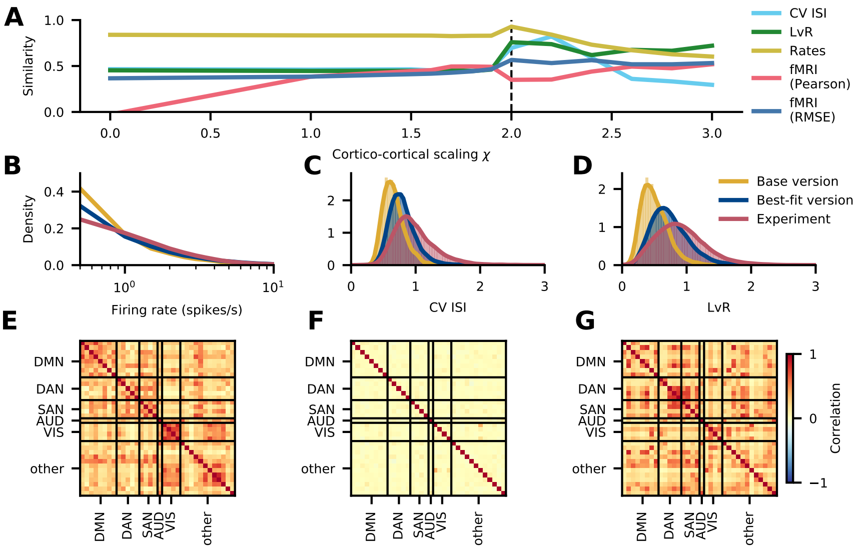

Fig. S6. Scaling plot using layer-resolved excitatory-to-inhibitory ratios of neuron densities from Gabbott, Somogyi (1986); Binzegger et al. (2004); Potjans, Diesmann (2014). The main difference compared to Shapson-Coe et al. (2024) is the higher fraction of excitatory cells in layer 2/3: 0.78 vs. 0.65. The other fractions are similar between the two studies: layer 4: 0.80 vs. 0.79, layer 5: 0.82 vs. 0.78, and layer 6: 0.83 vs. 0.86 for Binzegger et al. (2004); Potjans, Diesmann (2014) and Shapson-Coe et al. (2024), respectively. In this model realization, the additional scaling parameter χ_I for inter-areal connections onto inhibitory neurons is $\chi_I = 1$. The similarities of rates, CV ISI, LvR, and fMRI Pearson correlation stay roughly constant for inter-areal scaling factors $\chi < 2$. The best-fit version is shown as a dashed vertical line at $\chi = 2$. Here, the distributions of rates, CV ISI, and LvR match the experimental data well, and the fMRI RMSE is better than in the base version, while the fMRI Pearson correlation is slightly worse. For $\chi > 2$, the similarities of rates and CV ISI slowly deteriorate, while the fMRI Pearson correlation improves **(A)**. The match of the firing rates **(B)**, CV ISI **(C)** and LvR **(D)** to the experimental data is better in the best-fit version compared to the base version. The functional connectivity in the base version is uniformly weak **(F)**, while it is more differentiated in the best-fit version **(G)**.

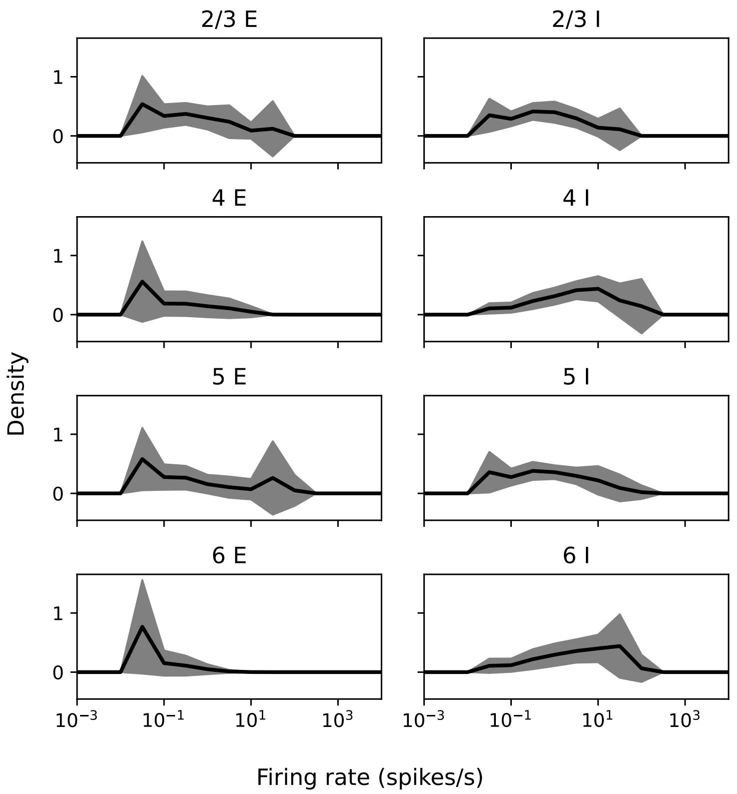

Fig. S7. Firing rate distribution density for the best-fit version ($\chi = 2.5$) in the original model, averaged across areas. The firing rate distribution densities of individual neurons were calculated for each population type (E and I) and layer (2/3, 4, 5, and 6) in a given area. Then, these distributions for each population were averaged across areas and plotted as a solid black line, with the standard deviation represented by the shaded region.

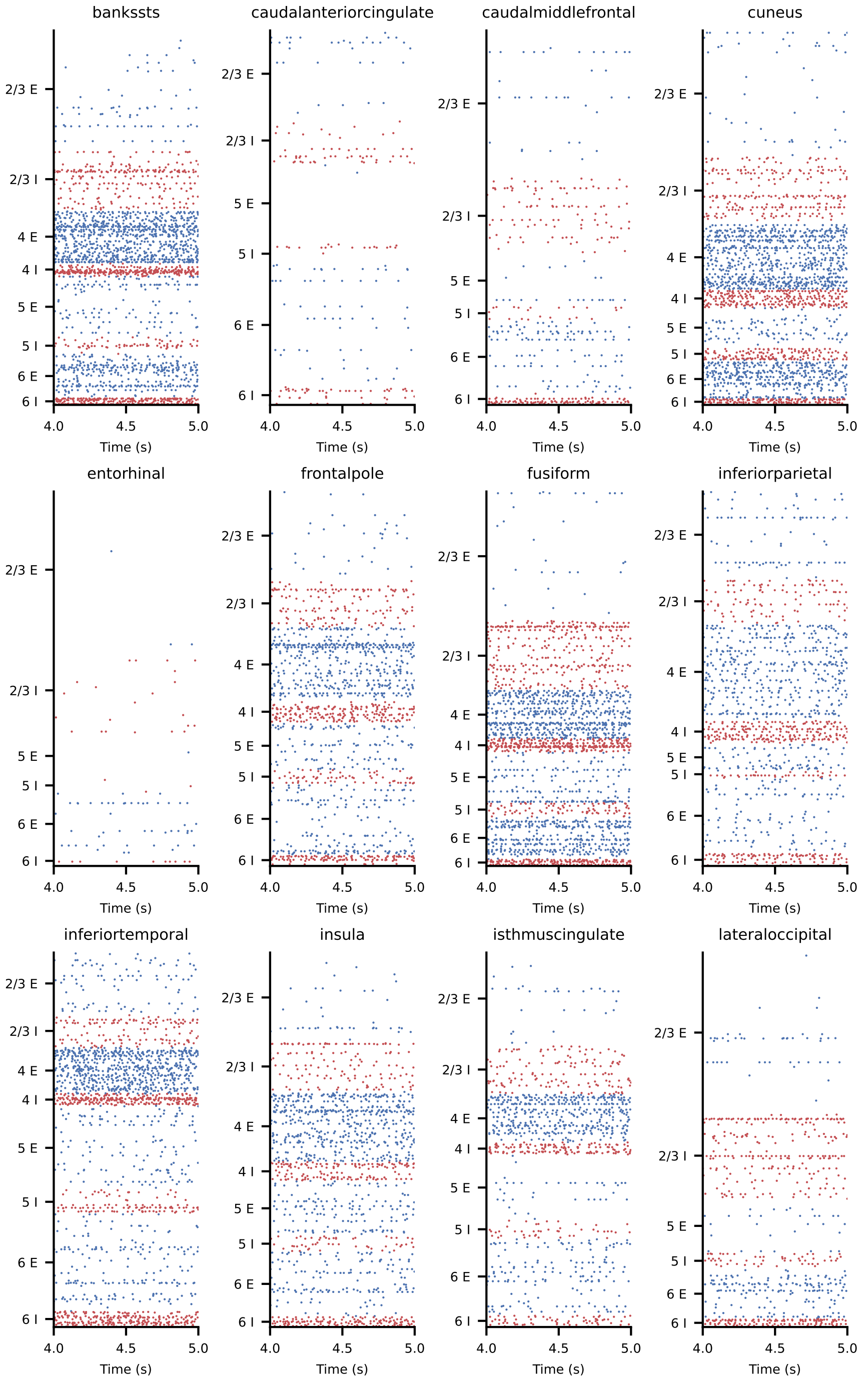

Fig. S8. Raster plots for the version without long-range inter-areal connections ($\chi = 0$). Subsampled to 2.5% of the excitatory (blue) and inhibitory (red) neurons.

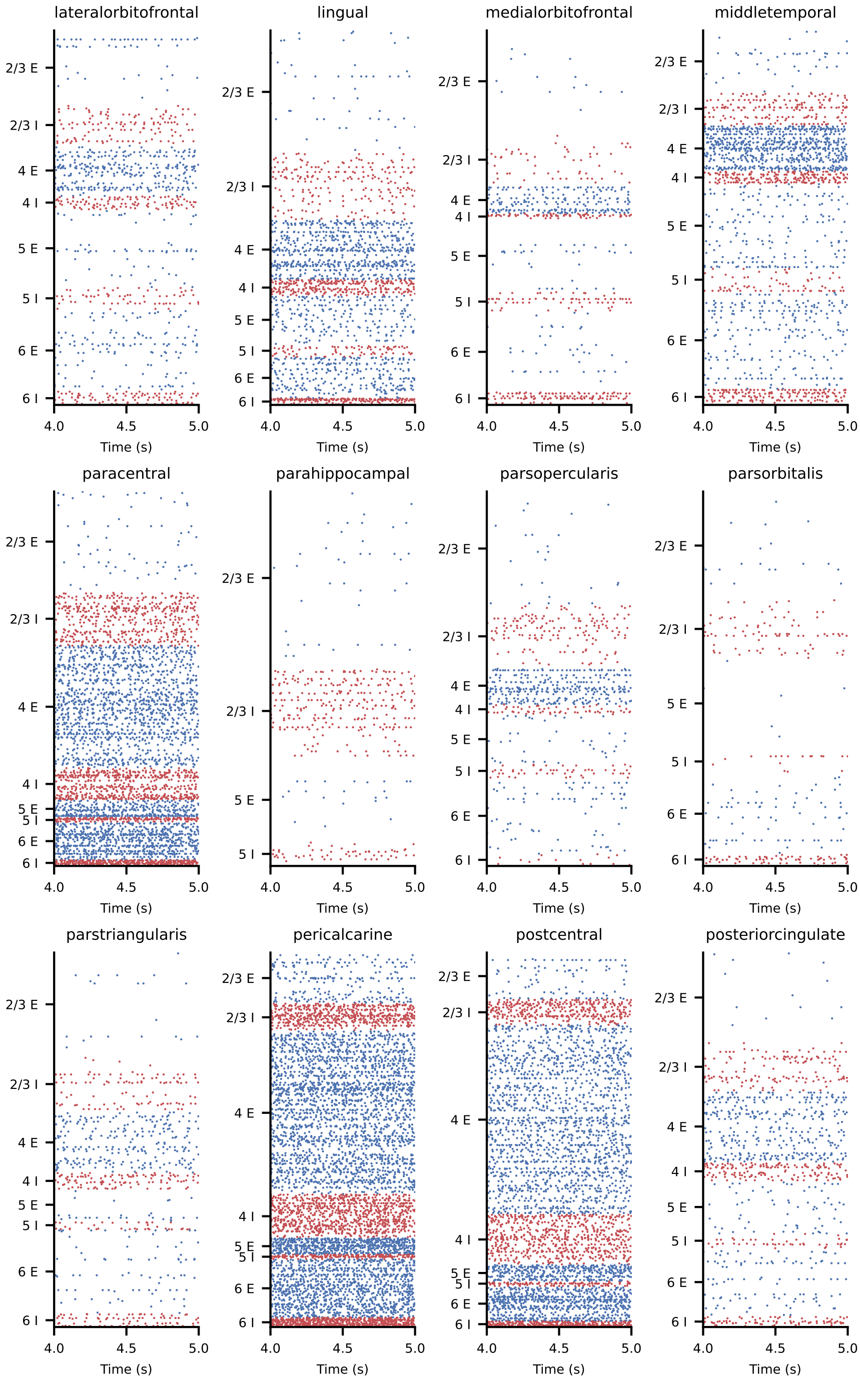

Fig. S9. Raster plots for the version without long-range inter-areal connections ($\chi = 0$). Subsampled to 2.5% of the excitatory (blue) and inhibitory (red) neurons.

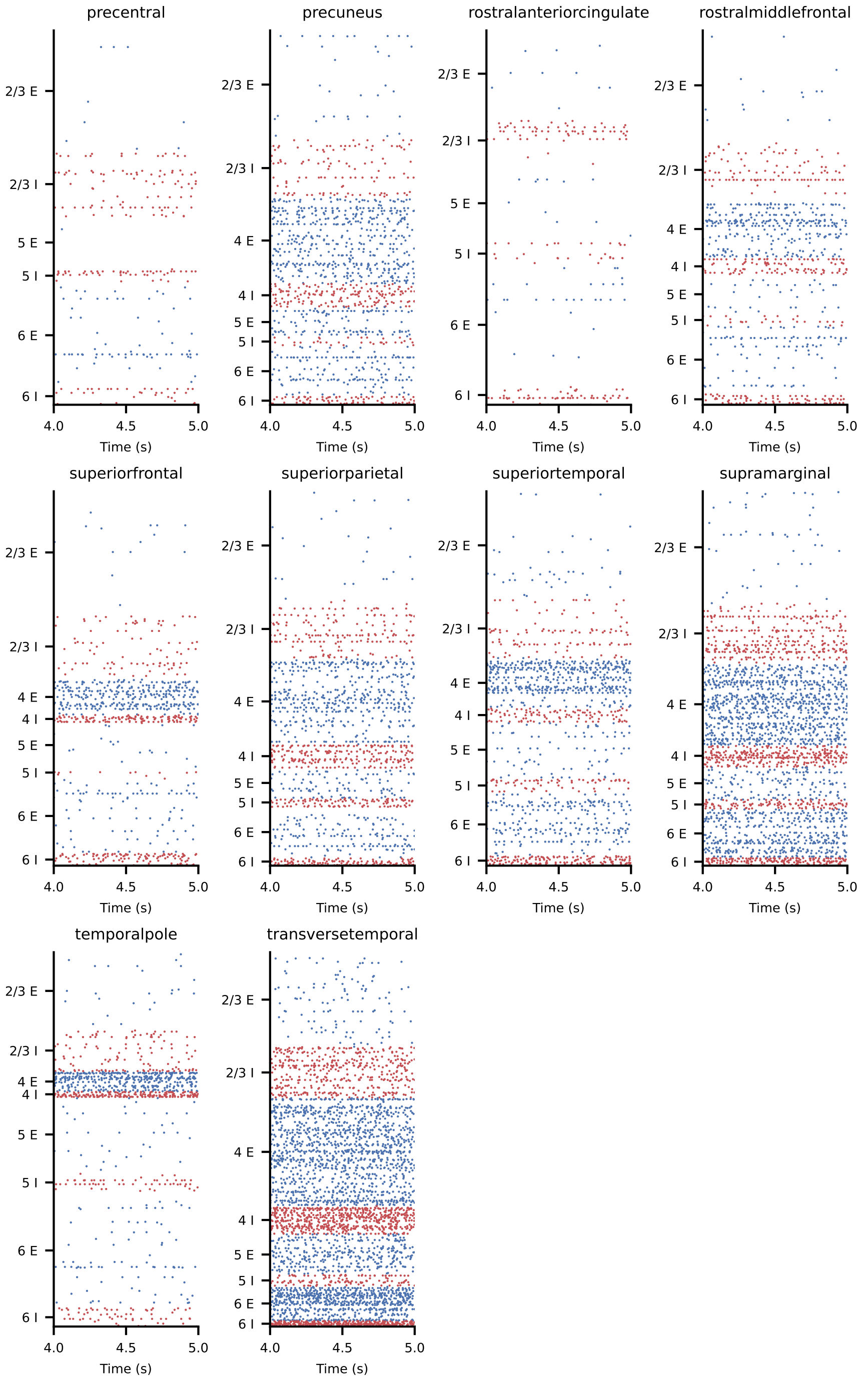

Fig. S10. Raster plots for the version without long-range inter-areal connections ($\chi = 0$). Subsampled to 2.5% of the excitatory (blue) and inhibitory (red) neurons.

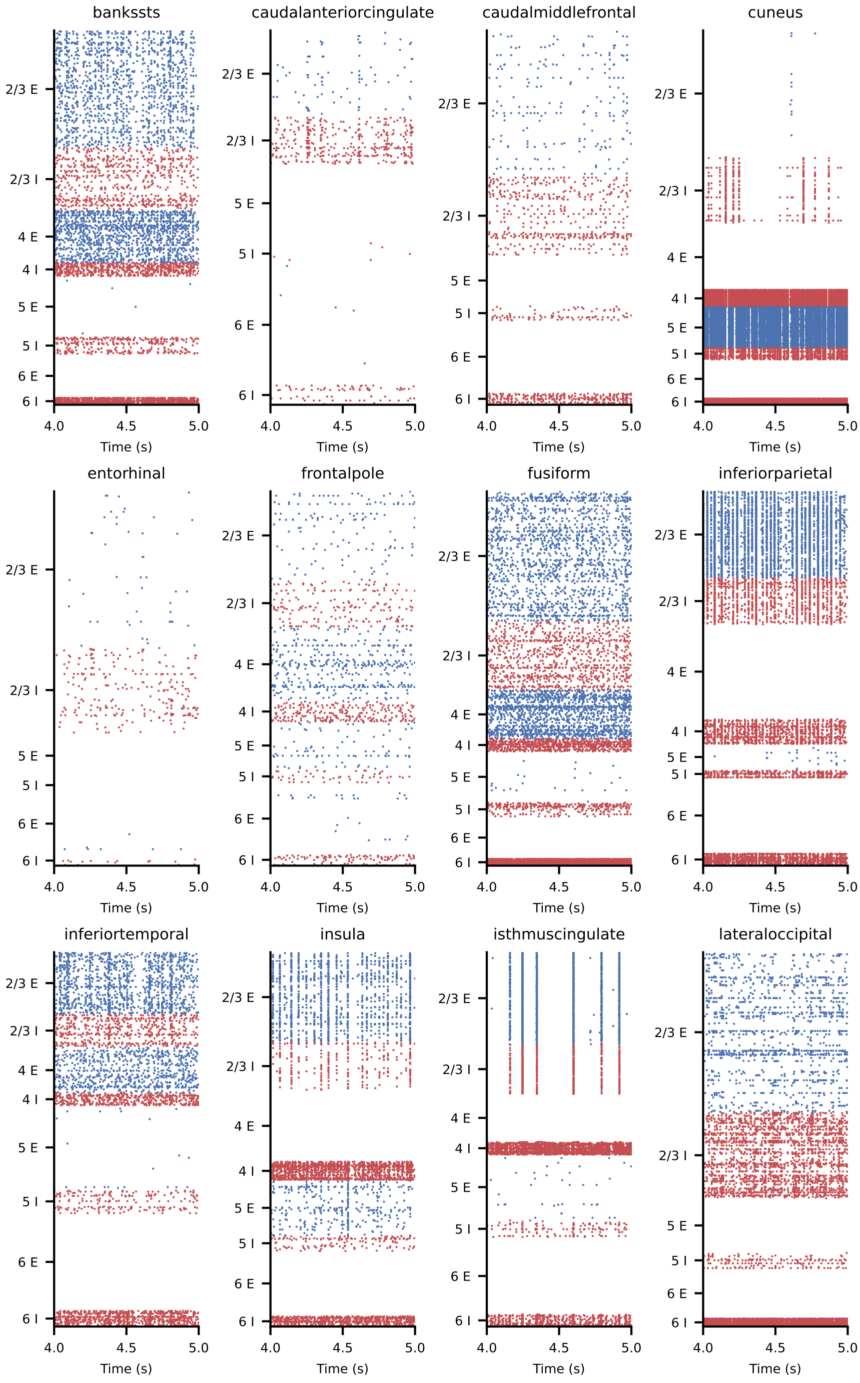

Fig. S11. Raster plots for the best-fit version. Subsampled to 2.5% of the excitatory (blue) and inhibitory (red) neurons.

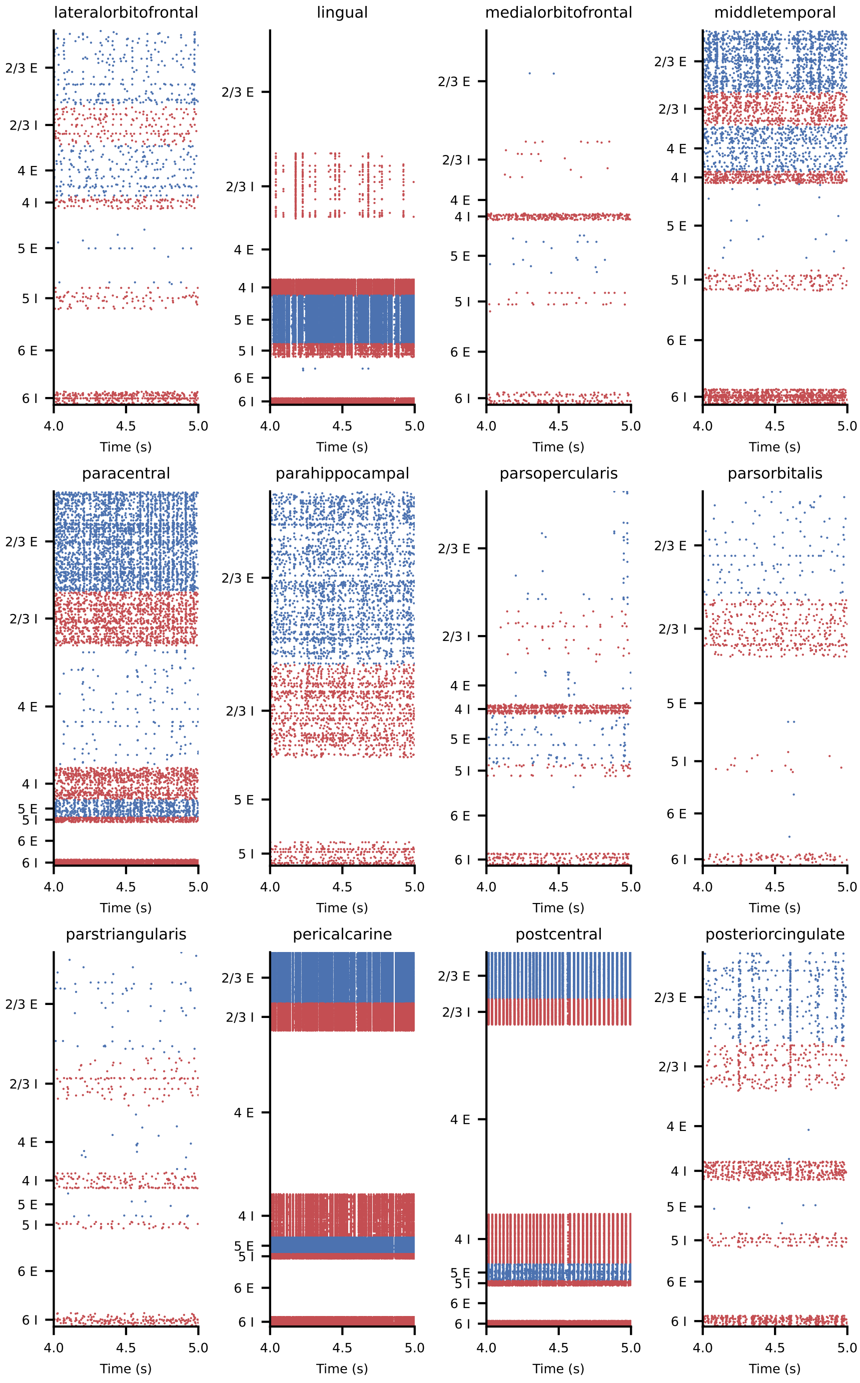

Fig. S12. Raster plots for the best-fit version. Subsampled to 2.5% of the excitatory (blue) and inhibitory (red) neurons.

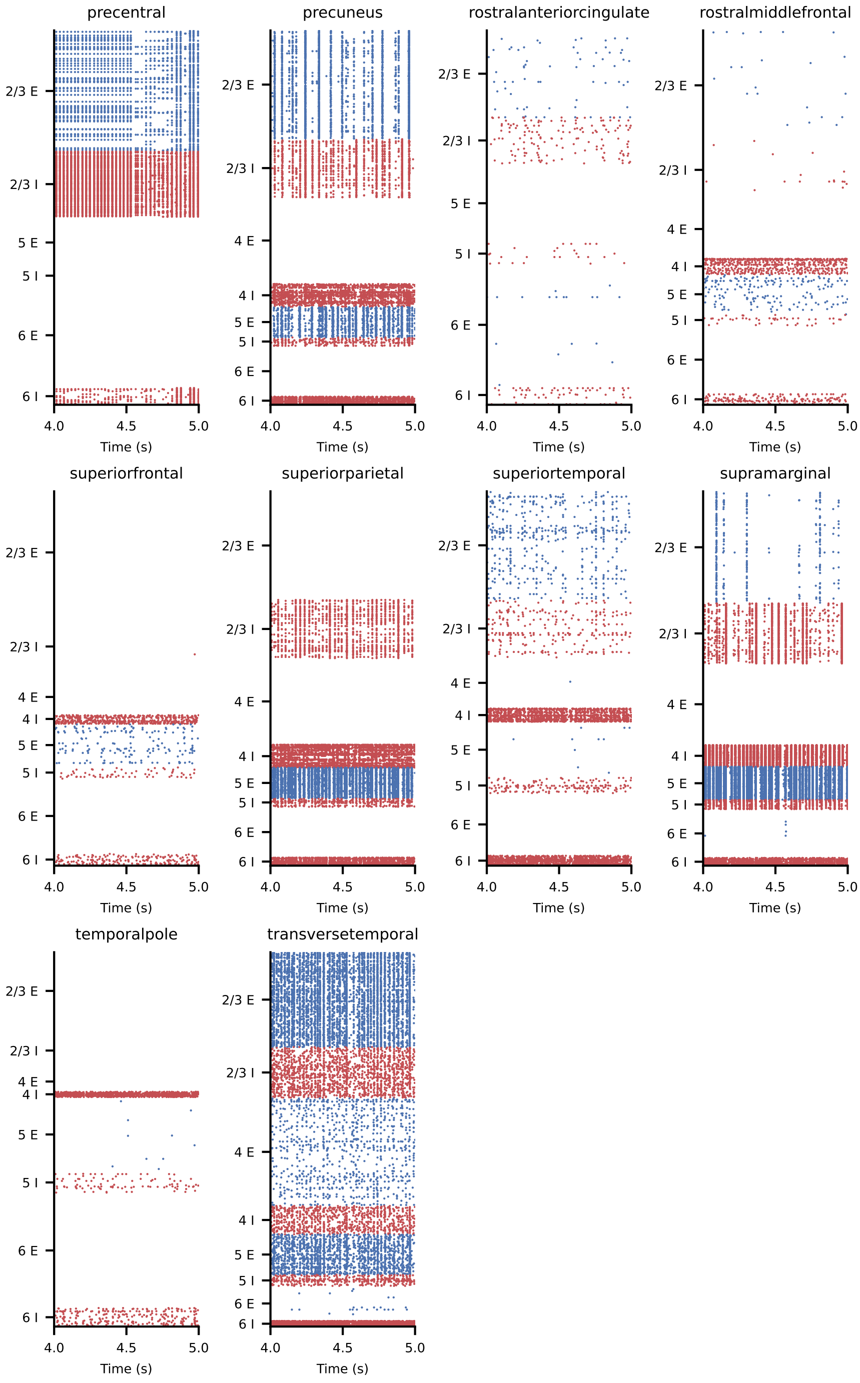

Fig. S13. Raster plots for the best-fit version. Subsampled to 2.5% of the excitatory (blue) and inhibitory (red) neurons.

Appendix References

Alonso-Nanclares L, Gonzalez-Soriano J, Rodriguez J, DeFelipe J. 2008. Gender differences in human cortical synaptic density. Proc Natl Acad Sci USA. *105*(38):14615–14619.

Amit DJ, Brunel N. 1997. Model of global spontaneous activity and local structured activity during delay periods in the cerebral cortex. Cereb Cortex. *7*:237–252.

Anderson JC, Martin KAC. 2009. The synaptic connections between cortical areas V1 and V2 in macaque monkey. J Neurosci. *29*(36):11283–11293.

Axer M, Grässel D, Kleiner M, Dammers J, Dickscheid T, Reckfort J, Hütz T, Eiben B, Pietrzyk U, Zilles K, et al. 2011. High-resolution fiber tract reconstruction in the human brain by means of three-dimensional polarized light imaging. Front Neuroinform. *5*:34.

Barbas H, Hilgetag CC, Saha S, Dermon CR, Suski JL. 2005. Parallel organization of contralateral and ipsilateral prefrontal cortical projections in the rhesus monkey. BMC Neurosci. *6*(1):32.

Berg J, Sorensen SA, Ting JT, Miller JA, Chartrand T, Buchin A, Bakken TE, Budzillo A, Dee N, Ding SL, Gouwens NW, Hodge RD, Kalmbach B, Lee C, Lee BR, Alfiler L, Baker K, Barkan E, Beller A, Berry K, Bertagnolli D, Bickley K, Bomben J, Braun T, Brouner K, Casper T, Chong P, Crichton K, Dalley R, de Frates R, Desta T, Lee SD, D'Orazi F, Dotson N, Egdorf T, Enstrom R, Farrell C, Feng D, Fong O, Furdan S, Galakhova AA, Gamlin C, Gary A, Glandon A, Goldy J, Gorham M, Goriounova NA, Gratiy S, Graybuck L, Gu H, Hadley K, Hansen N, Heistek TS, Henry AM, Heyer DB, Hill D, Hill C, Hupp M, Jarsky T, Kebede S, Keene L, Kim L, Kim MH, Kroll M, Latimer C, Levi BP, Link KE, Mallory M, Mann R, Marshall D, Maxwell M, McGraw M, McMillen D, Melief E, Mertens EJ, Mezei L, Mihut N, Mok S, Molnar G, Mukora A, Ng L, Ngo K, Nicovich PR, Nyhus J, Olah G, Oldre A, Omstead V, Ozsvar A, Park D, Peng H, Pham T, Pom CA, Potekhina L, Rajanbabu R, Ransford S, Reid D, Rimorin C, Ruiz A, Sandman D, Sulc J, Sunkin SM, Szafer A, Szemenyei V, Thomsen ER, Tieu M, Torkelson A, Trinh J, Tung H, Wakeman W, Waleboer F, Ward K, Wilbers R, Williams G, Yao Z, Yoon JG, Anastassiou C, Arkhipov A, Barzo P, Bernard A, Cobbs C, de Witt Hamer PC, Ellenbogen RG, Esposito L, Ferreira M, Gwinn RP, Hawrylycz MJ, Hof PR, Idema S, Jones AR, Keene CD, Ko AL, Murphy GJ, Ng L, Ojemann JG, Patel AP, Phillips JW, Silbergeld DL, Smith K, Tasic B, Yuste R, Segev I, de Kock CPJ, Mansvelder HD, Tamas G, Zeng H, Koch C, Lein ES. 2021. Human neocortical expansion involves glutamatergic neuron diversification. Nature. *598*(7879):151–158.

Binzegger T, Douglas RJ, Martin KAC. 2004. A quantitative map of the circuit of cat primary visual cortex. J Neurosci. *39*(24):8441–8453.

Boldog E, Bakken TE, Hodge RD, Novotny M, Aevermann BD, Baka J, Bordé S, Close JL, Diez-Fuertes F, Ding SL, et al. 2018. Transcriptomic and morphophysiological evidence for a specialized human cortical GABAergic cell type. Nat Neurosci. *21*(9):1185–1195.

Cano-Astorga N, DeFelipe J, Alonso-Nanclares L. 2021. Three-dimensional synaptic organization of layer III of the human temporal neocortex. Cereb Cortex. *31*(10):4742–4764.

Chartrand T, Dalley R, Close J, Goriounova NA, Lee BR, Mann R, Miller JA, Molnar G, Mukora A, Alfiler L, Baker K, Bakken TE, Berg J, Bertagnolli D, Braun T, Brouner K, Casper T, Csajbok EA, Dee N, Egdorf T, Enstrom R, Galakhova AA, Gary A, Gelfand E, Goldy J, Hadley K, Heistek TS, Hill D, Jorstad N, Kim L, Kocsis AK, Kruse L, Kunst M, Leon G, Long B, Mallory M, McGraw M, McMillen D, Melief EJ, Mihut N, Ng L, Nyhus J, Oláh G, Ozsvár A, Omstead V, Peterfi Z, Pom A, Potekhina L, Rajanbabu R, Rozsa M, Ruiz A, Sandle J, Sunkin SM, Szots I, Tieu M, Toth M, Trinh J, Vargas S, Vumbaco D, Williams G, Wilson J, Yao Z, Barzo P, Cobbs C, Ellenbogen RG, Esposito L, Ferreira M, Gouwens NW, Grannan B, Gwinn RP, Hauptman JS, Jarsky T, Keene CD, Ko AL, Koch C, Ojemann JG, Patel A, Ruzevick J, Silbergeld DL, Smith K, Sorensen SA, Tasic B, Ting JT, Waters J, de Kock CP, Mansvelder HD, Tamas G, Zeng H, Kalmbach B, Lein ES. 2023. Morphoelectric and transcriptomic divergence of the layer 1 interneuron repertoire in human versus mouse neocortex. Science. *382*(6667).

Colby C, Gattass R, Olson C, Gross C. 1988. Topographical organization of cortical afferents to extrastriate visual area PO in the macaque: a dual tracer study. J Comp Neurol. *269*(3):392–413.

Collins CE, Airey DC, Young NA, Leitch DB, Kaas JH. 2010. Neuron densities vary across and within cortical areas in primates. Proc Natl Acad Sci USA. *107*(36):15927–15932.

Cragg B. 1967. The density of synapses and neurones in the motor and visual areas of the cerebral cortex. J Anat. *101*(4):639–654.

DeFelipe J, Alonso-Nanclares L, Arellano J. 2002. Microstructure of the neocortex: comparative aspects. J Neurocytol. *31*:299–316.

Dehay C, Kennedy H, Bullier J, Berland M. 1988. Absence of interhemispheric connections of area 17 during development in the monkey. Nature. *331*(6154):348–350.

Douglas RJ, Martin KAC. 2004. Neuronal circuits of the neocortex. Annu Rev Neurosci. *27*:419–451.

Douglas RJ, Martin KAC, Whitteridge D. 1989. A canonical microcircuit for neocortex. Neural Comput. *1*(4):480–488.

Fabri M, Manzoni T. 1996. Glutamate decarboxylase immunoreactivity in corticocortical projecting neurons of rat somatic sensory cortex. Neuroscience. *72*(2):435–448.

Fabri M, Manzoni T. 2004. Glutamic acid decarboxylase immunoreactivity in callosal projecting neurons of cat and rat somatic sensory areas. Neuroscience. *123*(2):557–566.

Fourcaud N, Brunel N. 2002. Dynamics of the firing probability of noisy integrate-and-fire neurons. Neural Comput. *14*:2057–2110.

Gabbott PL, Somogyi P. 1986. Quantitative distribution of GABA-immunoreactive neurons in the visual cortex (area 17) of the cat. Exp Brain Res. *61*(2):323–331.

Galuske R, Schlote W, Bratzke H, Singer W. 2000. Interhemispheric asymmetries of the modular structure in human temporal cortex. Science. *5486*(289):1946–1949.

Gattass R, Sousa A, Mishkin M, Ungerleider L. 1997. Cortical projections of area V2 in the macaque. Cereb Cortex. *7*(2):110–129.

Girard P, Hupé JM, Bullier J. 2001. Feedforward and feedback connections between areas v1 and v2 of the monkey have similar rapid conduction velocities. J Neurophysiol. *85*(3):1328–1331.

Gonchar Y, Johnson P, Weinberg R. 1995. GABA-immunopositive neurons in rat neocortex with contralateral projections to SI. Brain Res. *697*(1):27–34.

Goulas A, Werner R, Beul SF, Säring D, Heuvel Mvd, Triarhou LC, Hilgetag CC. 2016. Cytoarchitectonic similarity is a wiring principle of the human connectome. BioRxiv.

Gouwens NW, Sorensen SA, Berg J, Lee C, Jarsky T, Ting J, Sunkin SM, Feng D, Anastassiou CA, Barkan E, et al. 2019. Classification of electrophysiological and morphological neuron types in the mouse visual cortex. Nat Neurosci. *22*:1182–1195.

Harrison KH, Hof PR, Wang SH. 2002. Scaling laws in the mammalian neocortex: Does form provide clues to function? J Neurocytol. *31*:289–298.

Herculano-Houzel S. 2009. The human brain in numbers: a linearly scaled-up primate brain. Front Hum Neurosci. *3*:31.

Herculano-Houzel S, Mota B, Wong P, Kaas JH. 2010. Connectivity-driven white matter scaling and folding in primate cerebral cortex. Proc Natl Acad Sci USA. *107*(44):19008–19013.

Higo S, Udaka N, Tamamaki N. 2007. Long-range GABAergic projection neurons in the cat neocortex. J Comp Neurol. *503*(3):421–431.

Hodge RD, Bakken TE, Miller JA, Smith KA, Barkan ER, Graybuck LT, Close JL, Long B, Johansen N, Penn O, Yao Z, Eggermont J, Höllt T, Levi BP, Shehata SI, Aevermann B, Beller A, Bertagnolli D, Brouner K, Casper T, Cobbs C, Dalley R, Dee N, Ding SL, Ellenbogen RG, Fong O, Garren E, Goldy J, Gwinn RP, Hirschstein D, Keene CD, Keshk M, Ko AL, Lathia K, Mahfouz A, Maltzer Z, McGraw M, Nguyen TN, Nyhus J, Ojemann JG, Oldre A, Parry S, Reynolds S, Rimorin C, Shapovalova NV, Somasundaram S, Szafer A, Thomsen ER, Tieu M, Quon G, Scheuermann RH, Yuste R, Sunkin SM, Lelieveldt B, Feng D, Ng L, Bernard A, Hawrylycz M, Phillips JW, Tasic B, Zeng H, Jones AR, Christof K, Lein ES. 2019. Conserved cell types with divergent features in human versus mouse cortex. Nature. *573*(7772):61–68.

Jiang HJ, Qi G, Duarte R, Feldmeyer D, van Albada SJ. 2023. A layered microcircuit model of somatosensory cortex with three interneuron types and cell-type-specific short-term plasticity. BioRxiv.:2023–10.

Jorstad NL, Close J, Johansen N, Yanny AM, Barkan ER, Travaglini KJ, Bertagnolli D, Campos J, Casper T, Crichton K, et al. 2023. Transcriptomic cytoarchitecture reveals principles of human neocortex organization. Science. *382*(6667):eadf6812.

Maksimov A, Diesmann M, van Albada SJ. 2018. Criteria on balance, stability, and excitability in cortical networks for constraining computational models. Front Comput Neurosci. *12*:44.

Markov NT, Ercsey-Ravasz MM, Ribeiro Gomes AR, Lamy C, Magrou L, Vezoli J, Misery P, Falchier A, Quilodran R, Gariel MA, Sallet J, Gamanut R, Huissoud C, Clavagnier S, Giroud P, Sappey-Marinier D, Barone P, Dehay C, Toroczkai Z, Knoblauch K, Van Essen DC, Kennedy H. 2014a. A weighted and directed interareal connectivity matrix for macaque cerebral cortex. Cereb Cortex. *24*(1):17–36.

Markov NT, Misery P, Falchier A, Lamy C, Vezoli J, Quilodran R, Gariel MA, Giroud P, Ercsey-Ravasz M, Pilaz LJ, Huissoud C, Barone P, Dehay C, Toroczkai Z, Van Essen DC, Kennedy H, Knoblauch K. 2011. Weight consistency specifies regularities of macaque cortical networks. Cereb Cortex. *21*(6):1254–1272.

Markov NT, Vezoli J, Chameau P, Falchier A, Quilodran R, Huissoud C, Lamy C, Misery P, Giroud P, Ullman S, Barone P, Dehay C, Knoblauch K, Kennedy H. 2014b. Anatomy of hierarchy: Feedforward and feedback pathways in macaque visual cortex. J Comp Neurol. *522*(1):225–259.

Markram H, Muller E, Ramaswamy S, Reimann MW, Abdellah M, Sanchez CA, Ailamaki A, Alonso-Nanclares L, Antille N, Arsever S, Kahou GAA, Berger TK, Bilgili A, Buncic N, Chalimourda A, Chindemi G, Courcol JD, Delalondre F, Delattre V, Druckmann S, Dumusc R, Dynes J, Eilemann S, Gal E, Gevaert ME, Ghobril JP, Gidon A, Graham JW, Gupta A, Haenel V, Hay E, Heinis T, Hernando JB, Hines M, Kanari L, Keller D, Kenyon J, Khazen G, Kim Y, King JG, Kisvarday Z, Kumbhar P, Lasserre S, Bé JVL, Magalhães BR, Merchán-Pérez A, Meystre J, Morrice BR, Muller J, Muñoz-Céspedes A, Muralidhar S, Muthurasa K, Nachbaur D, Newton TH, Nolte M, Ovcharenko A, Palacios J, Pastor L, Perin R, Ranjan R, Riachi I, Rodríguez JR, Riquelme JL, Rössert C, Sfyrakis K, Shi Y, Shillcock JC, Silberberg G, Silva R, Tauheed F, Telefont M, Toledo-Rodriguez M, Tränkler T, Geit WV, Díaz JV, Walker R, Wang Y, Zaninetta SM, DeFelipe J, Hill SL, Segev I, Schürmann F. 2015. Reconstruction and simulation of neocortical microcircuitry. Cell. *163*(2):456–492.

McDonald CT, Burkhalter A. 1993. Organisation of long-range inhibitory connections within rat visual cortex. J Neurosci. *13*(2):768–781.

Miller DJ, Bhaduri A, Sestan N, Kriegstein A. 2019. Shared and derived features of cellular diversity in the human cerebral cortex. Curr Opin Neurobiol. *56*:117–124.

Minxha J, Adolphs R, Fusi S, Mamelak AN, Rutishauser U. 2020. Flexible recruitment of memory-based choice representations by the human medial frontal cortex. Science. *368*(6498):eaba3313.

Mohan H, Verhoog MB, Doreswamy KK, Eyal G, Aardse R, Lodder BN, Goriounova NA, Asamoah B, B. Brakspear AC, Groot C, van der Sluis S, Testa-Silva G, Obermayer J, Boudewijns ZS, Narayanan RT, Baayen JC, Segev I, Mansvelder HD, de Kock CP. 2015. Dendritic and axonal architecture of individual pyramidal neurons across layers of adult human neocortex. Cereb Cortex. *25*(12):4839–4853.

Mohan H, Verhoog MB, Doreswamy KK, Eyal G, Aardse R, Lodder BN, Goriounova NA, Asamoah B, Brakspear ACB, Groot C, Van Der Sluis S, Testa-Silva G, Obermayer J, Boudewijns ZS, Narayanan RT, Baayen JC, Segev I, Mansvelder HD, De Kock CP. 2023. Morphological data of human neocortical pyramidal neurons (v1.1) (v1.1).

O'Kusky J, Colonnier M. 1982. A laminar analysis of the number of neurons, glia, and synapses in the visual cortex (area 17) of adult macaque monkeys. J Comp Neurol. *210*(3):278–290.

Packer AM, Yuste R. 2011. Dense, unspecific connectivity of neocortical parvalbumin-positive interneurons: A canonical microcircuit for inhibition? J Neurosci. *31*(37):13260–13271.

Perin R, Berger TK, Markram H. 2011. A synaptic organizing principle for cortical neuronal groups. Proc Natl Acad Sci USA. *108*(13):5419–5424.

Pinto A, Fuentes C, Paré D. 2006. Feedforward inhibition regulates perirhinal transmission of neocortical inputs to the entorhinal cortex: ultrastructural study in guinea pigs. J Comp Neurol. *495*(6):722–734.

Potjans TC, Diesmann M. 2014. The cell-type specific cortical microcircuit: Relating structure and activity in a full-scale spiking network model. Cereb Cortex. *24*(3):785–806.

Reimann MW, Horlemann AL, Ramaswamy S, Muller EB, Markram H. 2017. Morphological diversity strongly constrains synaptic connectivity and plasticity. Cereb Cortex. *27*(9):4570–4585.

Rosen BQ, Halgren E. 2022. An estimation of the absolute number of axons indicates that human cortical areas are sparsely connected. PLOS Biol. *20*(3):e3001575.

Ruddy KL, Leemans A, Carson RG. 2017. Transcallosal connectivity of the human cortical motor network. Brain Struct Funct. *222*:1243–1252.

Salin P, Bullier J, Kennedy H. 1989. Convergence and divergence in the afferent projections to cat area 17. J Comp Neurol. *283*(4):486–512.

Schmidt M, Bakker R, Hilgetag CC, Diesmann M, van Albada SJ. 2018. Multi-scale account of the network structure of macaque visual cortex. Brain Struct Funct. *223*(3):1409–1435.

Schuman B, Dellal S, Prönneke A, Machold R, Rudy B. 2021. Neocortical layer 1: An elegant solution to top-down and bottom-up integration. Annual Review of Neuroscience. *44*(1):221–252.

Seehaus AK, Roebroeck A, Chiry O, Kim DS, Ronen I, Bratzke H, Goebel R, Galuske RA. 2013. Histological validation of DW-MRI tractography in human postmortem tissue. Cereb Cortex. *23*(2):442–450.

Senk J, Hagen E, van Albada SJ, Diesmann M. 2023. Reconciliation of weak pairwise spike-train correlations and highly coherent local field potentials across space. ArXiv.:1805.10235v1 [q–bio.NC].

Shapson-Coe A, Januszewski M, Berger DR, Pope A, Wu Y, Blakely T, Schalek RL, Li PH, Wang S, Maitin-Shepard J, et al. 2024. A petavoxel fragment of human cerebral cortex reconstructed at nanoscale resolution. Science. *384*(6696):eadk4858.

Sherwood CC, Miller SB, Karl M, Stimpson CD, Phillips KA, Jacobs B, Hof PR, Raghanti MA, Smaers JB. 2020. Invariant synapse density and neuronal connectivity scaling in primate neocortical evolution. Cereb Cortex. *30*(10):5604–5615.

Stephan K, Kamper L, Bozkurt A, Burns G, Young M, Kötter R. 2001. Advanced database methodology for the collation of connectivity data on the macaque brain (CoCoMac). Philos Trans R Soc B. *356*:1159–1186.

Swindale NV, Spacek MA, Krause M, Mitelut C. 2023. Spontaneous activity in cortical neurons is stereotyped and non-poisson. Cereb Cortex. *33*(11):6508–6525.

Tardif E, Clarke S. 2001. Intrinsic connectivity of human auditory areas: a tracing study with DiI. Eur J Neurosci. *13*(5):1045–1050.

Teeter C, Iyer R, Menon V, Gouwens N, Feng D, Berg J, Szafer A, Cain N, Zeng H, Hawrylycz M, Koch C, Mihalas S. 2018. Generalized leaky integrate-and-fire models classify multiple neuron types. Nat Commun. *9*:709.

Tomioka R, Okamoto K, Furuta T, Fujiyama F, Iwasato T, Yanagawa Y, Obata K, Kaneko T, Tamamaki N. 2005. Demonstration of long-range GABAergic connections distributed throughout the mouse neocortex. Eur J Neurosci. *21*:1587–1600.

Tomioka R, Rockland KS. 2007. Long-distance corticocortical GABAergic neurons in the adult monkey white and gray matter. J Comp Neurol. *505*(5):526–538.

Urrutia-Piñones J, Morales-Moraga C, Sanguinetti-González N, Escobar AP, Chiu CQ. 2022. Long-range GABAergic projections of cortical origin in brain function. Front Syst Neurosci. *16*:841869.

Van Essen DC, Smith SM, Barch DM, Behrens TE, Yacoub E, Ugurbil K, Consortium WMH, et al. 2013. The wu-minn human connectome project: An overview. Neuroimage. *80*:62–79.

Vandevelde I, Duckworth E, Reep R. 1996. Layer VII and the gray matter trajectories of corticocortical axons in rats. Anatomy and Embryology. *194*:581–593.

Von Economo C. 2009. *Cellular Structure of the Human Cerebral Cortex*. Karger Medical and Scientific Publishers. Translated and edited by L.C. Triarhou.

Wagstyl K, Larocque S, Cucurull G, Lepage C, Cohen JP, Bludau S, Palomero-Gallagher N, Lewis LB, Funck T, Spitzer H, et al. 2020. Bigbrain 3D atlas of cortical layers: Cortical and laminar thickness gradients diverge in sensory and motor cortices. PLOS Biol. *18*(4):e3000678.

Winnubst J, Bas E, Ferreira TA, Wu Z, Economo MN, Edson P, Arthur BJ, Bruns C, Rokicki K, Schauder D, et al. 2019. Reconstruction of 1,000 projection neurons reveals new cell types and organization of long-range connectivity in the mouse brain. Cell. *179*(1):268–281.
