# Supplementary material for: Multi-scale spiking network model of human cerebral cortex
Source: Cereb Cortex. 2024 Oct 18;34(10):bhae409. doi: 10.1093/cercor/bhae409 (PMC11491286; doi:10.1093/cercor/bhae409)
Supplement: HumanMultiScaleModel_latex [file humanmultiscalemodel_latex.zip › figs/figure_model_overview.pdf]

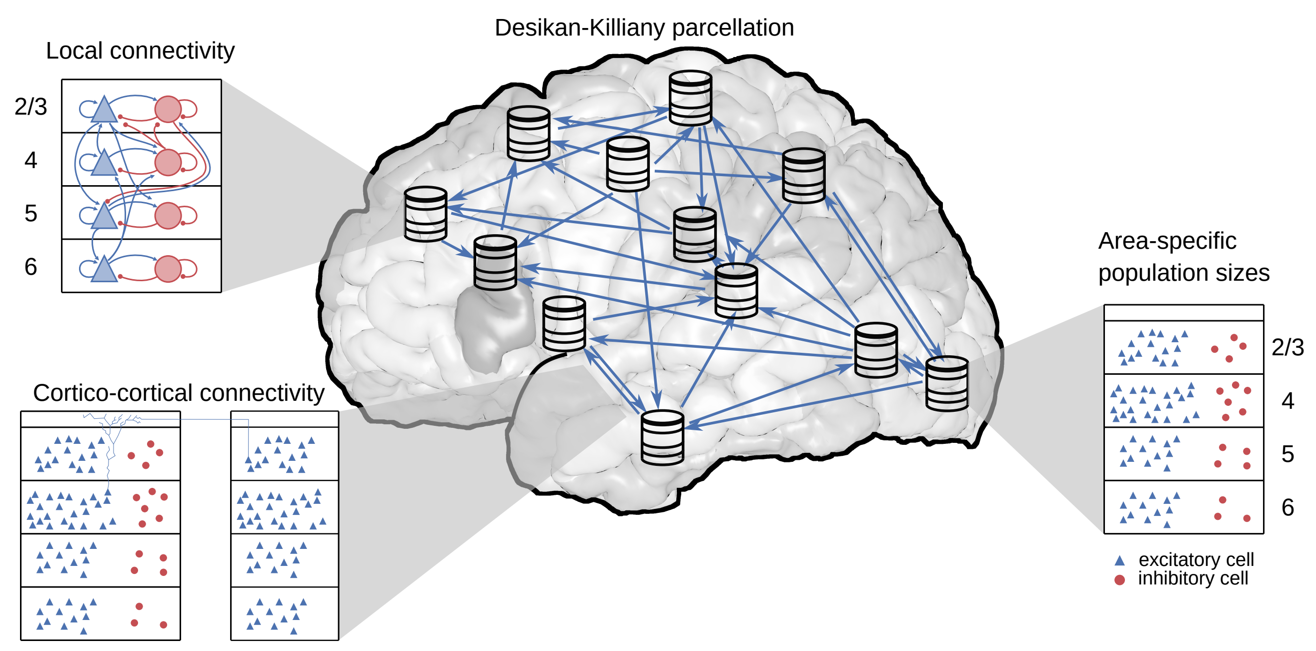

Desikan-Killiany parcellation
Local connectivity
2/3
4
5
6
Area-specific
population sizes
2/3
4
5
6
Cortico-cortical connectivity
excitatory cell
inhibitory cell
