# Supplementary figures and images for: Multi-scale spiking network model of human cerebral cortex
Source: Cereb Cortex. 2024 Oct 18;34(10):bhae409. doi: 10.1093/cercor/bhae409 (PMC11491286; doi:10.1093/cercor/bhae409)

A

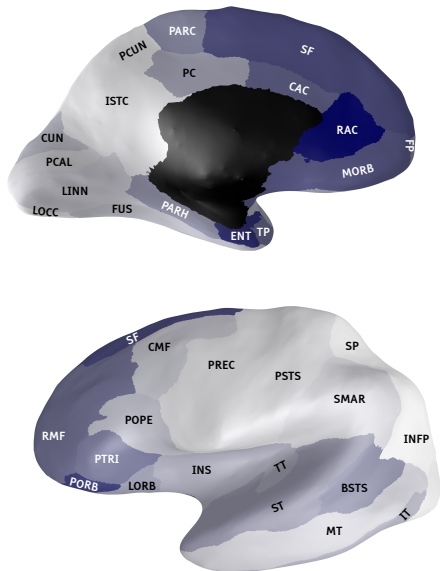

B

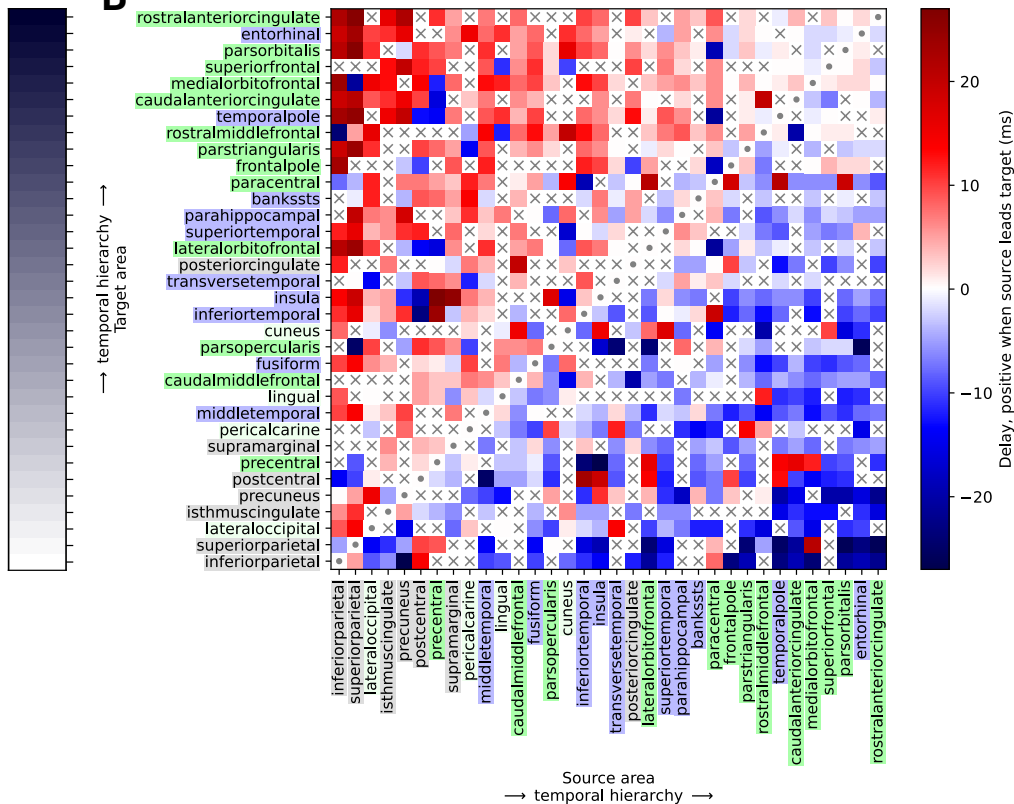

Supplement: HumanMultiScaleModel_latex [file humanmultiscalemodel_latex.zip › figs/figure_temporal_hierarchy.pdf]

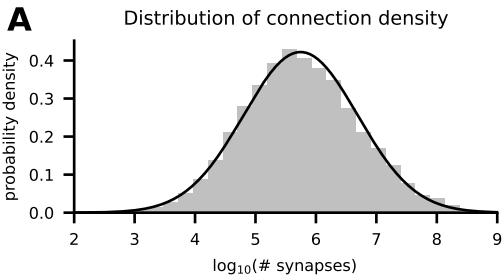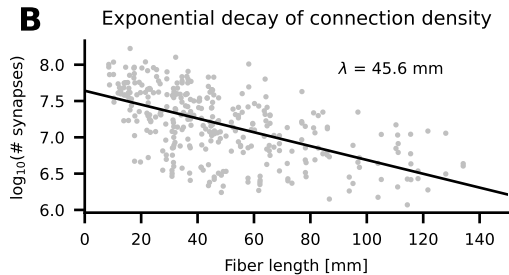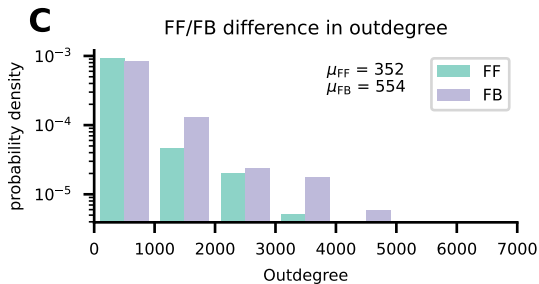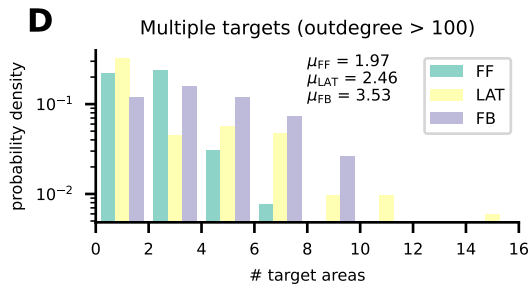

Supplement: HumanMultiScaleModel_latex [file humanmultiscalemodel_latex.zip › figs/figure_connectivity_validation.pdf]

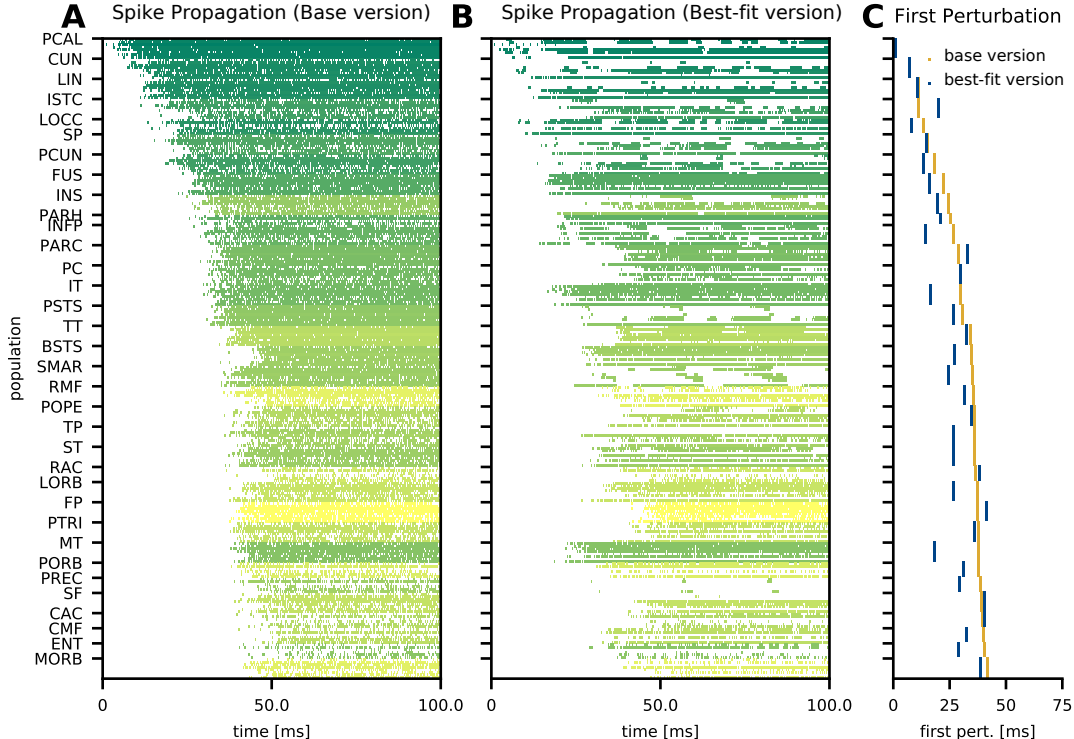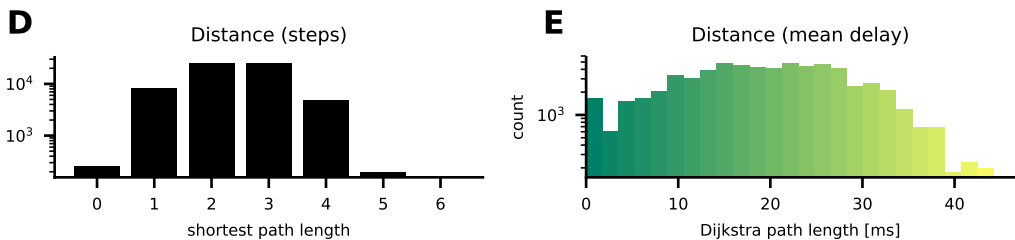

Supplement: HumanMultiScaleModel_latex [file humanmultiscalemodel_latex.zip › figs/figure_spike_journey.pdf]

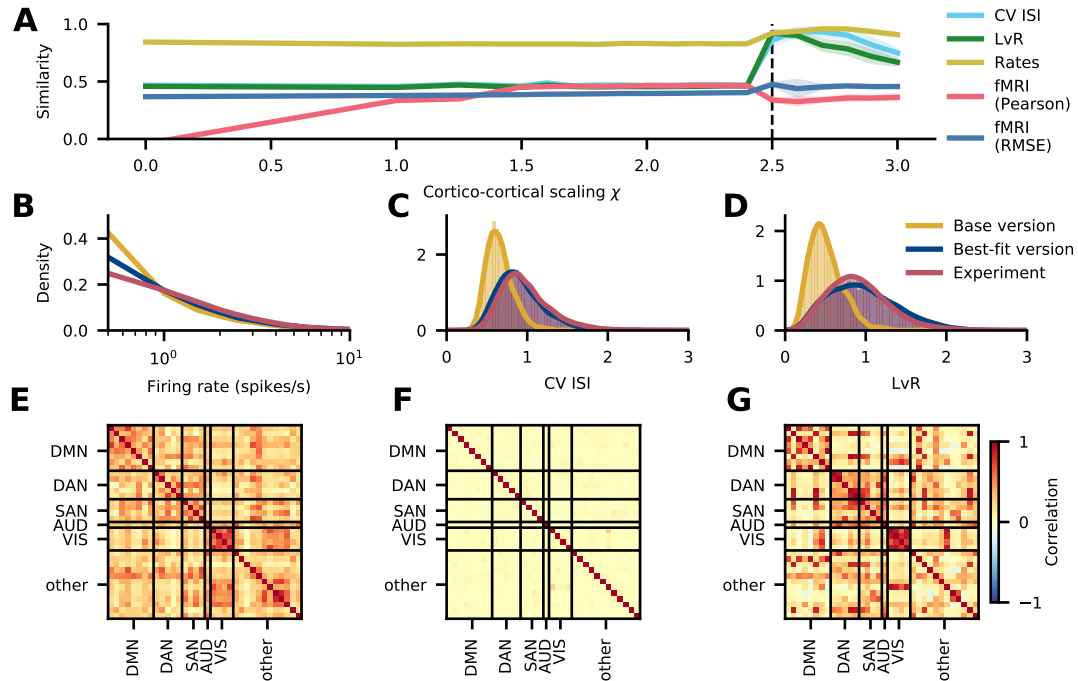

Supplement: HumanMultiScaleModel_latex [file humanmultiscalemodel_latex.zip › figs/figure_scaling_experiment_lichtman_chiI2_different_seed.pdf]

**A** caudalanteriorcingulate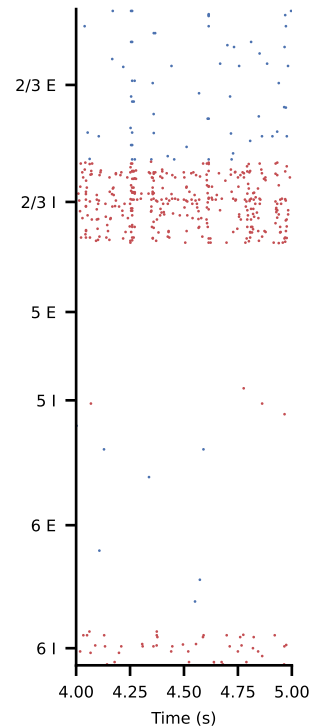**B** pericalcarine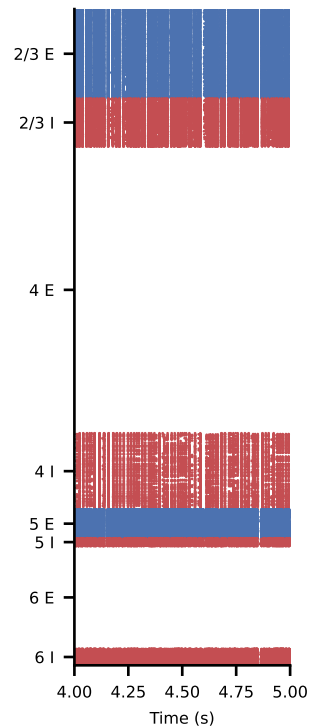**C** fusiform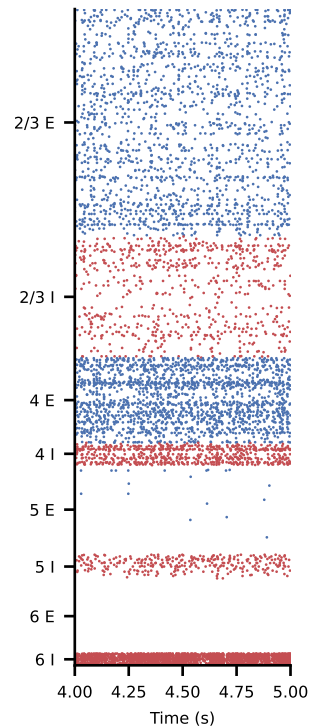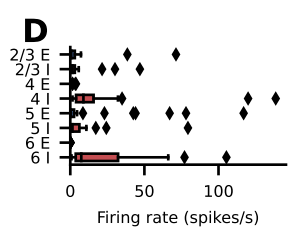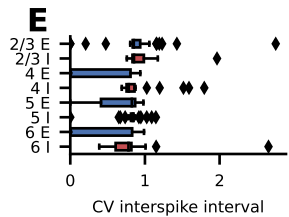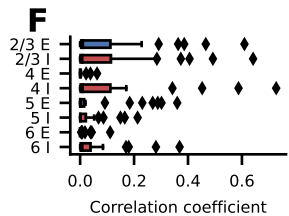

Supplement: HumanMultiScaleModel_latex [file humanmultiscalemodel_latex.zip › figs/figure_spike_statistics_metastable_lichtman_chiI2_different_seed.pdf]

**A** caudalanteriorcingulate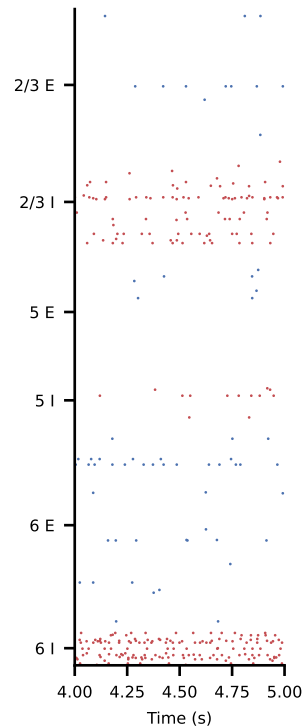**B** pericalcarine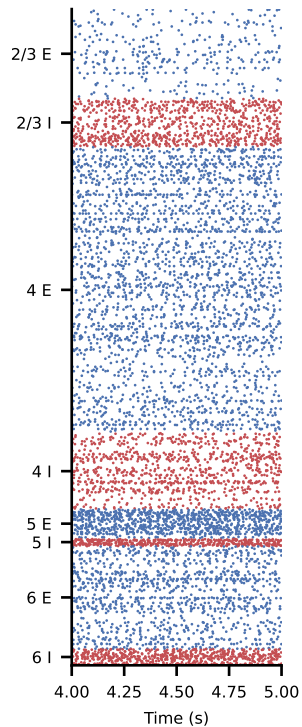**C** fusiform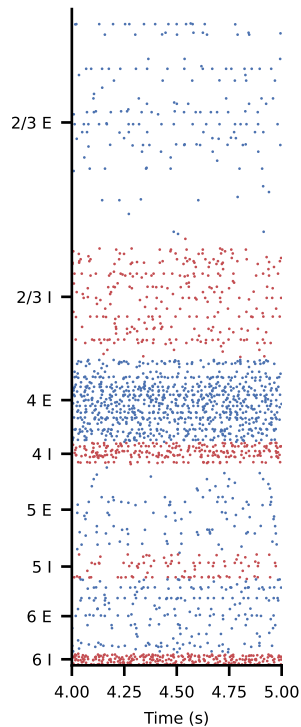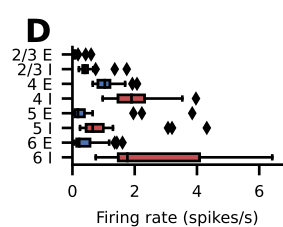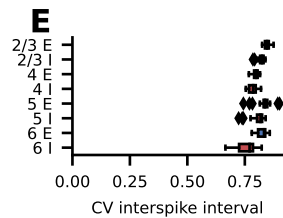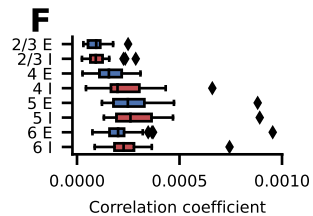

Supplement: HumanMultiScaleModel_latex [file humanmultiscalemodel_latex.zip › figs/figure_spike_statistics_groundstate_lichtman_chiI2_different_seed.pdf]

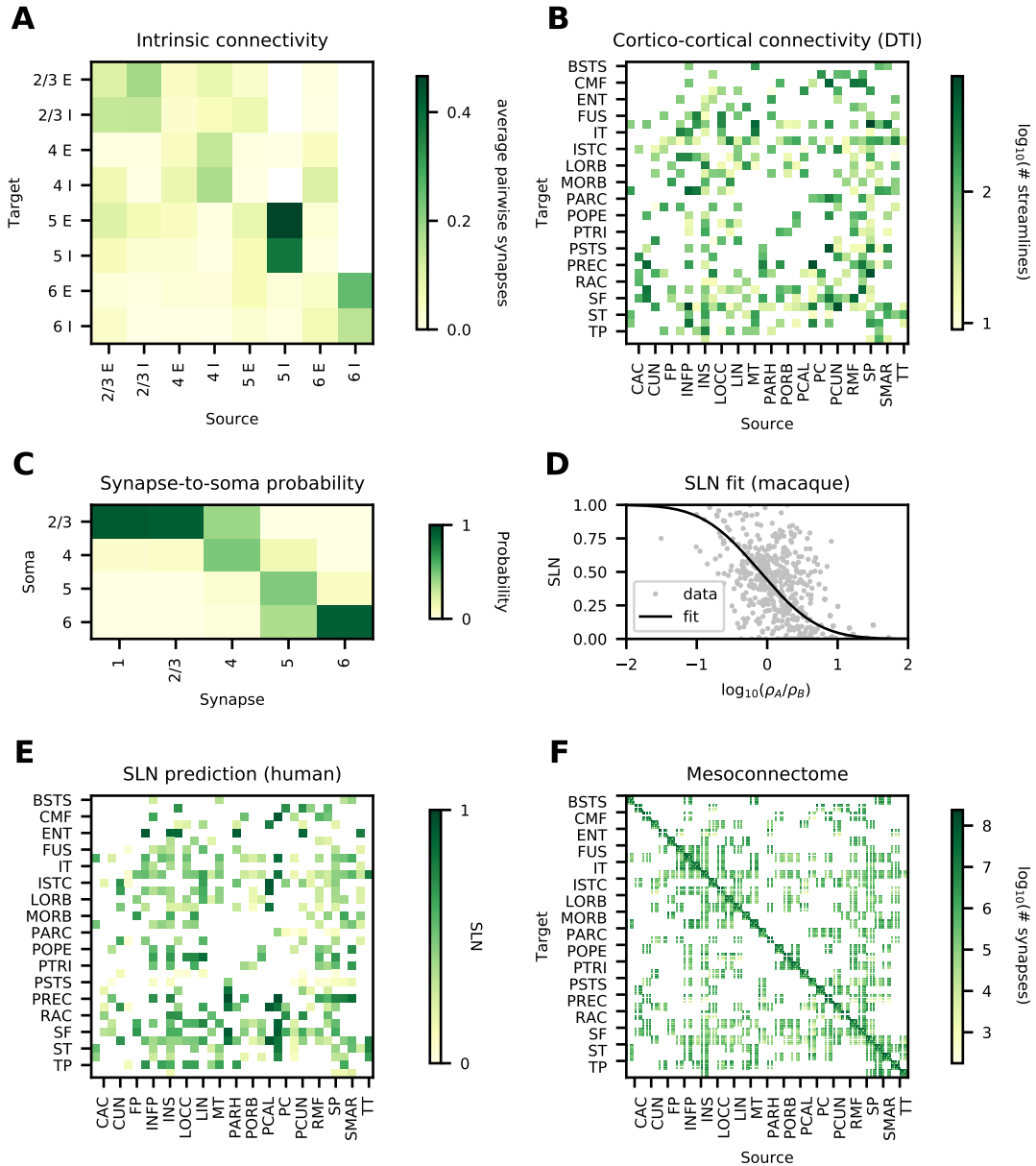

Supplement: HumanMultiScaleModel_latex [file humanmultiscalemodel_latex.zip › figs/figure_connectivity_construction.pdf]
